# Annually and monthly resolved solar irradiance and atmospheric temperature data across the Hawaiian archipelago from 1998 to 2015 with interannual summary statistics

**DOI:** 10.1016/j.dib.2018.05.099

**Published:** 2018-05-23

**Authors:** Richard Bryce, Ignacio Losada Carreño, Andrew Kumler, Bri-Mathias Hodge, Billy Roberts, Carlo Brancucci Martinez-Anido

**Affiliations:** a National Renewable Energy Laboratory, Golden, CO, United States; b Department of Mechanical and Industrial Engineering, University of Massachusetts, Amherst, MA, United States; c Department of Mechanical Engineering, Northern Arizona University, Flagstaff, AZ, United States

## Abstract

This article contains data and summary statistics of solar irradiance and dry bulb temperature across the Hawaiian archipelago resolved on a monthly basis and spanning years 1998–2015. This data was derived in association with an article titled “Consequences of Neglecting the Interannual Variability of the Solar Resource: A Case Study of Photovoltaic Power Among the Hawaiian Islands” (Bryce et al., 2018 [7]). The solar irradiance data is presented in terms of Direct Normal Irradiance (DNI), Diffuse Horizontal Irradiance (DHI), and Global Horizontal Irradiance (GHI) and was obtained from the satellite-derived data contained in the National Solar Radiation Database (NSRDB). The temperature data is also obtained from this source. We have processed the NSRDB data and compiled these monthly resolved data sets, along with interannual summary statistics including the interannual coefficient of variability.

## Specifications Table

**Table:** 

Subject area	*Solar Energy Engineering*
More specific subject area	*Variability of Solar Irradiance and Atmospheric temperature*
Type of data	*Graphical, Tabulated*
How data was acquired	*Processed data from the National Solar Radiation Database*
Data format	*Analyzed*
Experimental factors	
Experimental features	
Data source location	*Hawaiian Archipelago*
Data accessibility	*Analyzed data contained herein, source data available publicly from the National Solar Radiation Database, which can be accessed at*https://maps.nrel.gov/nsrdb-viewer/
Related research article	Bryce, R., Losada, I., Hodge, B., Martinez-anido, C.B., 2018. Consequences of Neglecting the Interannual Variability of the Solar Resource: A Case Study of Photovoltaic Power Among the Hawaiian Islands. Sol. Energy in press.

## Value of the data

• High penetration levels of solar renewable energy is enabled by accurate knowledge of the quantity and variability of the solar resource at a given location.
• Given objectives laid out by the Hawaiian Clean Energy Initiative which commits the state to generating 100% of the electrical energy consumed across the state by renewable energy sources by the year 2045 [1], knowledge of variability of the solar resource and temperature is essential for project planning and for stakeholders alike.
• Data contained herein allows for comparison of ground-based measurements of solar irradiance and temperature on the Hawaiian Islands against these satellite-derived values.
• Data contained herein allows for comparison between solar irradiance and dry-bulb temperature across Hawaii with other Pacific island chains.

## 1 Data

The U.S. Department of Energy has funded the multiyear production of the National Solar Radiation Database (NSRDB). This database has been updated three times since the initial public release in 1992. The most recent version contains a wide range of solar irradiance data derived from satellite data and meteorological data derived from reanalysis data. This data is presented with a 4-km by 4-km spatial resolution and half-hourly temporal resolution covering the 18 years from 1998 to 2015 [2]. The reanalysis data is obtained from NASA׳s Modern-Era Retrospective Analysis for Research and Applications (MERRA) data set [3]. This version of the NSRDB includes data from the area bounded by the 25°W and 175°W meridians and by the − 20°S and 60°N parallels.

The performance of the irradiance data sets in the most recent version of the NSRDB was evaluated in [2]; the hourly average satellite-derived data have a mean bias error of ± 5% for GHI and less than ± 10% for DNI when compared against concurrent ground-based measurements from seven sites of the Surface Radiation Budget Network. The NSRDB has been shown to accurately feature interannual variability; the coefficient of variation (COV) for both the satellite-derived data sets and ground-measured from seven Surface Radiation Budget Network stations across the United States where compared. The data sets show similar annual trends and differ by only 0.68% COV for global horizontal irradiance (GHI) and 0.97% COV for direct normal irradiance (DNI).

In the original public release, [3] reported the root mean squared residuals between the MERRA data sets and measured data. The MERRA data sets have also been independently evaluated in [4], [5]6]; the data sets feature a mean bias of dry-bulb temperature of less than 1 °C globally.

Herein, we share the monthly averaged solar irradiance and dry-bulb temperature data across all eight major islands of the Hawaiian Archipelago for all years from 1998 to 2015. We report box-plots of values from all the NSRDB grid cells, as well as tabulated island-wide mean, for each island. Along with the tabulated data, we report interannual summary statistics to quantify the interannual variability of solar irradiance, reported as GHI, and temperature for each island. This data and the summary statistics were compiled alongside another work which aimed to characterize the interannual variability of the solar irradiance and meteorological conditions across the State of Hawaii and to then characterize the variability of PV power generation [7].

## 2 Experimental design, materials, and methods

In compiling the data tables of GHI and atmospheric temperature, we sample 1-h resolution DNI, DHI, GHI, and temperature data among the Hawaiian Islands for the 18 years available in the NSRDB (1998–2015). Therefore, the basis data set we analyzed contained 8760 hourly values for the various grid cells over land of each island of Hawaii; 10 cells across Ni’ihau, 81 cells across Kauai, 82 cells across O’ahu, 35 cells across Moloka’i, 18 grid cells across Lanai, 7 cells across Kaho’olawe, 102 cells across Maui, and 559 cells across Hawai’i.

**Fig. 1:**
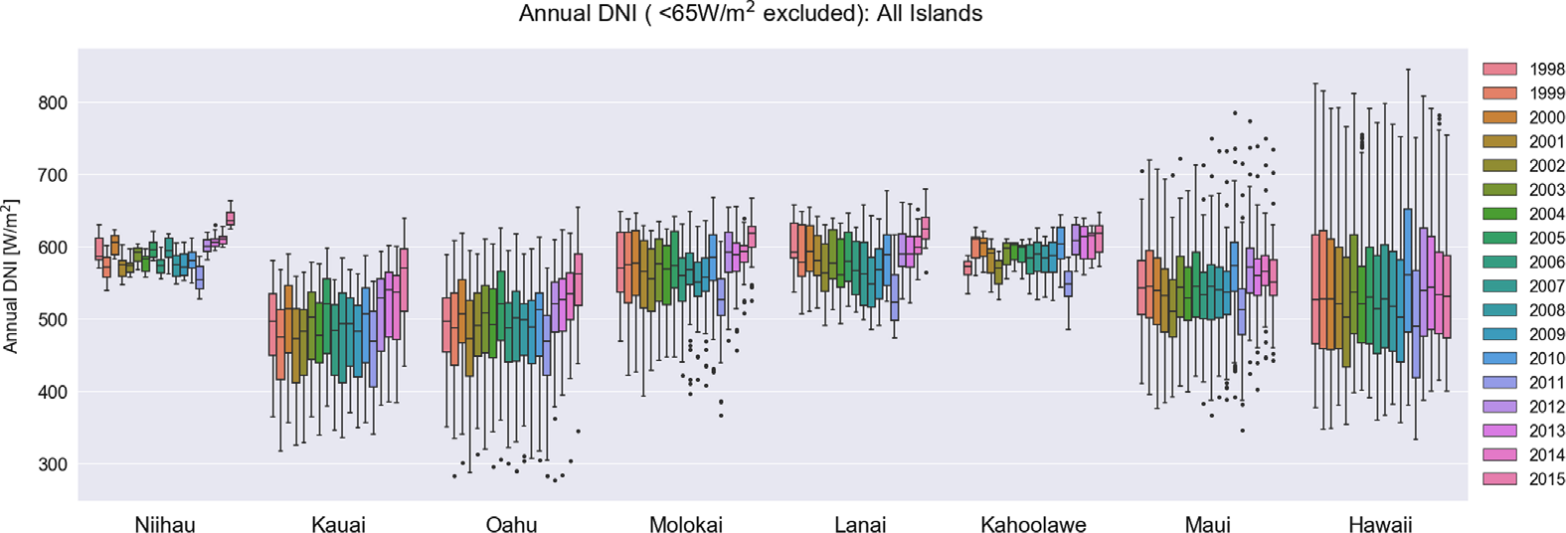
Box-plots of annual mean direct normal irradiance among all NSRDB grid cells sites for the years 1998–2015, repeated for each island of the Hawaiian archipelago. Outliers beyond 150% of the interquartile range are shown as dots.

**Fig. 2:**
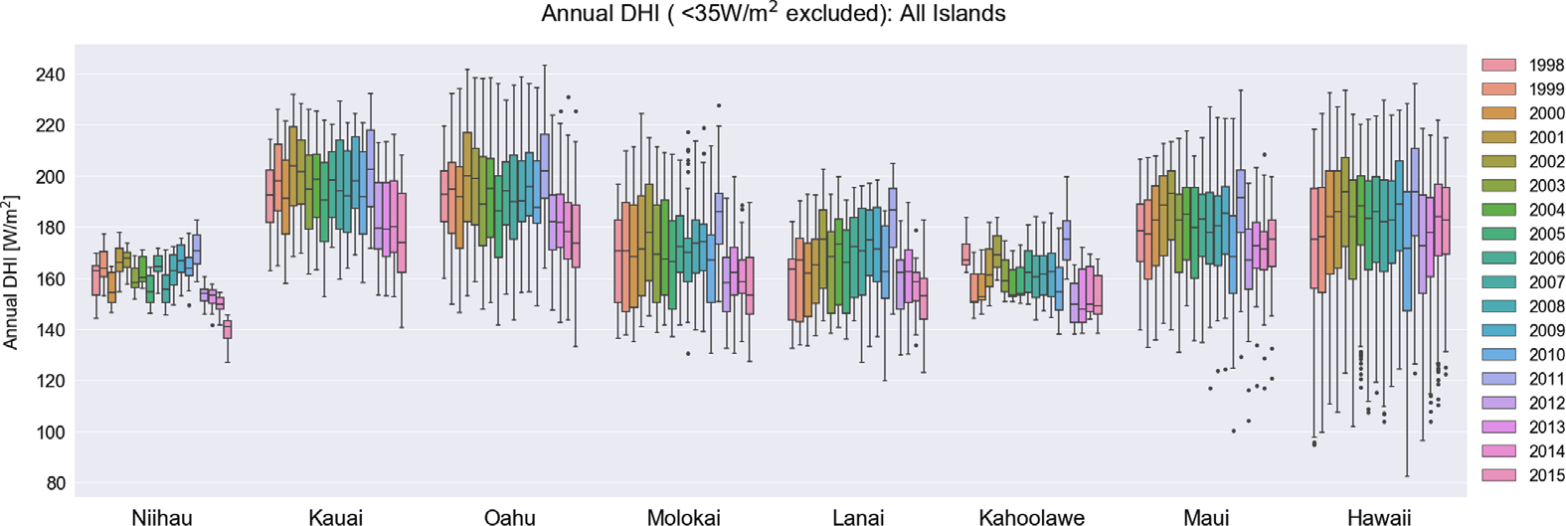
Box-plots of annual mean diffuse horizontal irradiance among all NSRDB grid cells sites for the years 1998–2015, repeated for each island of the Hawaiian archipelago. Outliers beyond 150% of the interquartile range are shown as dots.

**Fig. 3:**
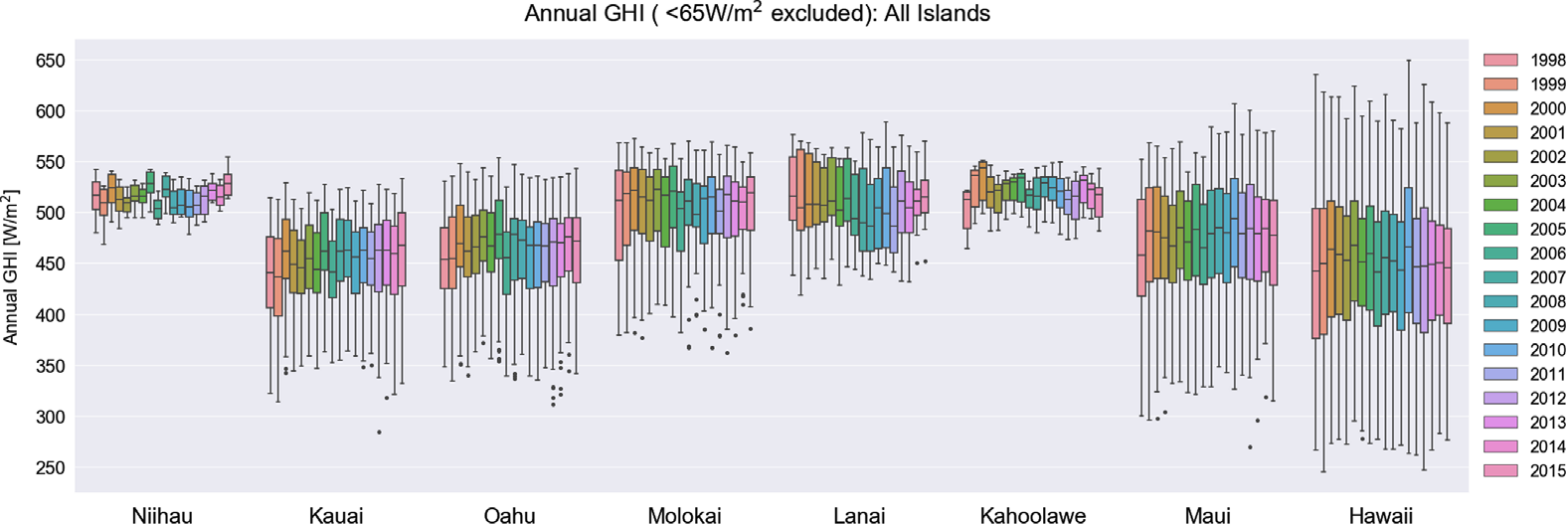
Box-plots of annual mean global normal irradiance among all NSRDB grid cells sites for the years 1998–2015, repeated for each island of the Hawaiian archipelago. Outliers beyond 150% of the interquartile range are shown as dots.

**Fig. 4:**
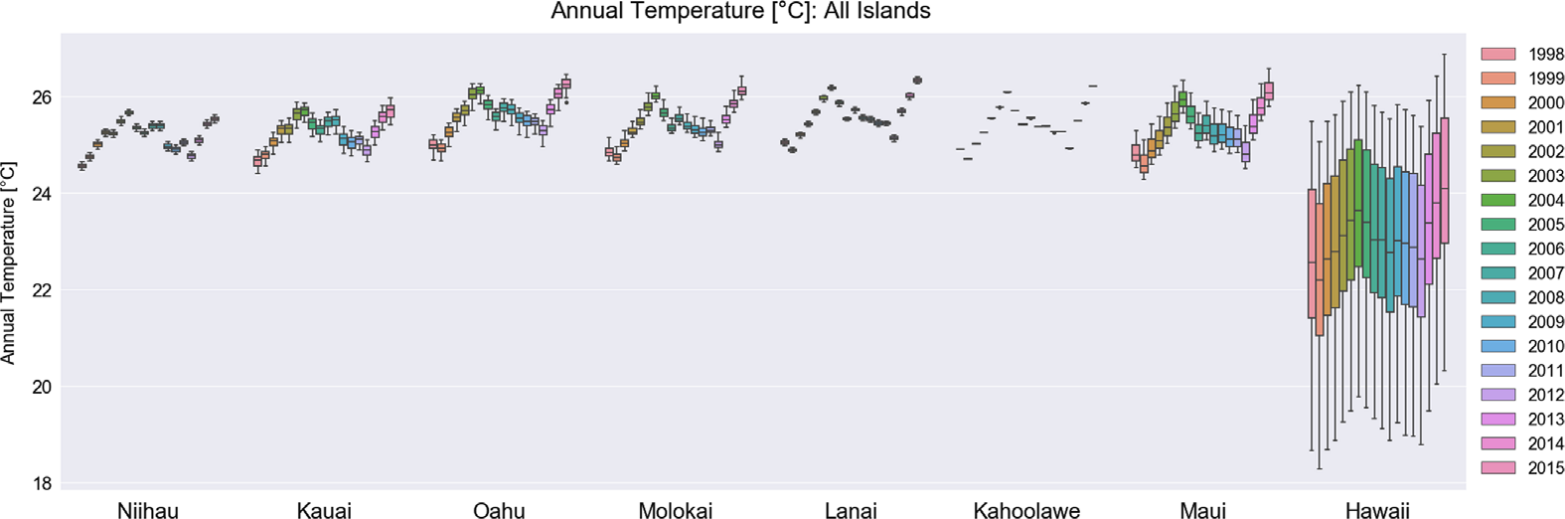
Box-plots of annual mean dry-bulb temperature (°C) among all NSRDB grid cells sites for the years 1998–2015, repeated for each island of the Hawaiian archipelago. Outliers beyond 150% of the interquartile range are shown as dots.

Solar irradiance values are often reported in a way that excludes values below a certain threshold of the clear-sky radiation [8]. This filter excludes the shading effect that is pronounced during dawn and dusk. To identify the appropriate threshold, we calculated the first nonzero irradiance value during each day for each site across the State of Hawaii; the mean value for GHI and DNI was 65 W/m^2^, whereas a lower value of 35 W/m^2^ for DHI was identified. No filter is applied to the temperature data. For all NSRDB cells across each island, the annual mean value was calculated. Then, a box plot of the annual mean value of all NSRDB cells was produced for each year of data (Fig. 1, Fig. 2, Fig. 3, Fig. 4). The data that was used to generate these box plots, including the minimum values, the values of the first quartiles, the values of the second quartiles, the values of the third quartiles, and the maximum annual mean values are tabulated in Table 1, Table 2, Table 3, Table 4.

**Table 1:** The minimum values, the quartile values, and the maximum annual mean values for DNI across each island during each year from 1998 to 2015.

		**1998**	**1999**	**2000**	**2001**	**2002**	**2003**	**2004**	**2005**	**2006**	**2007**	**2008**	**2009**	**2010**	**2011**	**2012**	**2013**	**2014**	**2015**
**Niihau**	**Min**	569.9	539.2	582.6	547.0	557.7	568.4	557.1	580.6	555.1	561.6	548.2	553.4	549.5	527.7	581.7	594.2	598.3	624.2
	**Q1**	576.5	554.0	588.0	556.1	562.5	576.1	562.7	583.3	561.0	579.5	553.8	558.0	565.1	539.7	589.1	597.5	600.6	628.8
	**Q2**	583.5	563.3	595.2	566.6	569.7	586.1	576.6	589.3	570.5	587.7	568.6	564.4	578.2	542.9	595.6	602.8	607.2	632.5
	**Q3**	609.9	579.4	612.0	579.3	576.3	595.1	585.4	603.2	578.8	605.8	579.4	588.9	585.8	568.5	606.1	607.2	613.4	642.8
	**Max**	629.8	601.6	623.1	602.8	596.7	603.9	596.1	620.4	598.3	617.6	604.7	606.0	611.1	586.7	619.7	629.7	627.3	662.6
**Kauai**	**Min**	365.2	317.4	356.8	325.4	328.6	364.2	339.9	379.5	346.8	335.7	370.1	349.3	357.0	340.9	380.3	385.7	384.0	434.8
	**Q1**	449.3	408.5	445.4	410.3	420.0	441.1	437.9	450.7	421.7	410.5	433.4	414.2	435.6	404.8	454.4	473.3	470.7	504.2
	**Q2**	495.0	473.7	513.6	470.9	482.1	494.6	475.9	518.1	481.1	492.1	489.5	482.3	504.9	463.3	526.3	535.2	534.0	568.1
	**Q3**	534.6	511.1	545.0	513.4	512.7	535.4	520.8	552.7	519.0	535.3	528.2	517.5	542.3	509.8	553.7	563.6	559.3	595.6
	**Max**	580.3	568.4	589.9	558.9	564.0	578.0	575.4	597.8	553.5	583.3	568.0	562.2	586.9	551.2	593.5	600.9	599.6	639.4
**Oahu**	**Min**	351.1	283.8	301.4	288.1	313.0	319.7	296.1	305.8	300.4	289.6	304.0	307.1	305.4	282.8	277.4	284.8	303.4	345.3
	**Q1**	452.6	434.4	462.7	417.7	448.2	452.3	444.7	468.9	440.0	439.2	448.2	433.0	446.9	421.1	481.2	482.7	497.2	516.6
	**Q2**	496.4	484.7	505.4	471.9	488.8	505.5	489.2	516.0	486.0	496.5	497.4	487.8	512.9	467.4	519.8	523.9	533.9	559.8
	**Q3**	523.7	531.7	552.9	518.3	533.4	545.6	538.7	561.8	521.8	537.9	520.7	518.9	531.0	503.5	549.9	552.0	561.9	589.1
	**Max**	588.9	608.1	618.2	594.5	589.1	610.5	603.5	625.3	593.8	617.4	589.2	596.1	612.4	570.0	609.1	622.7	618.8	654.1
**Molokai**	**Min**	469.2	421.9	427.0	393.8	429.2	443.2	447.3	447.8	422.2	396.3	416.0	408.0	426.8	367.0	470.0	456.5	507.7	524.8
	**Q1**	530.5	517.5	529.2	512.6	508.4	521.3	508.9	538.7	513.7	533.9	528.7	529.4	548.6	497.0	557.1	560.0	575.7	593.9
	**Q2**	566.2	563.4	576.2	554.2	551.4	564.0	567.8	566.1	553.7	566.4	548.7	556.7	584.4	526.1	591.2	588.1	592.9	615.9
	**Q3**	617.6	619.0	620.9	606.2	593.9	606.5	597.8	618.6	582.3	585.4	573.7	579.8	613.4	545.6	617.9	603.2	599.9	624.6
	**Max**	648.2	637.3	645.6	628.8	622.2	634.3	623.8	640.8	630.3	648.2	627.3	635.1	667.7	606.4	653.5	654.7	639.5	666.3
**Lanai**	**Min**	537.0	507.5	509.9	515.1	490.5	512.4	493.6	517.1	509.5	499.3	485.5	491.4	523.8	473.5	528.1	522.1	554.5	564.2
	**Q1**	573.0	554.1	564.6	553.7	530.7	554.1	538.0	546.2	527.3	516.0	508.9	527.3	541.7	494.9	571.9	569.8	587.7	606.4
	**Q2**	590.3	575.7	581.5	574.6	558.4	575.2	557.3	576.1	553.4	538.9	531.3	556.5	572.5	503.2	587.9	584.9	595.4	620.0
	**Q3**	631.1	629.2	622.8	612.2	602.8	620.4	608.7	619.3	598.6	594.0	582.9	586.2	615.3	557.8	616.1	601.4	610.2	638.6
	**Max**	656.8	648.4	654.4	641.7	630.1	638.7	631.8	645.5	626.2	657.1	641.8	638.1	677.1	616.3	660.7	658.5	651.0	679.0
**Kahoolawe**	**Min**	534.8	559.6	564.0	536.7	526.0	554.9	566.0	555.0	534.4	526.2	529.9	525.5	543.4	484.8	564.9	560.4	570.6	572.7
	**Q1**	551.5	561.5	567.3	546.3	535.4	561.5	567.7	557.6	554.8	550.8	555.2	552.4	569.3	517.8	571.5	573.8	579.2	585.3
	**Q2**	565.3	606.6	604.4	577.8	558.6	586.4	594.3	598.5	563.7	571.5	565.3	567.6	588.5	532.2	602.4	587.2	582.8	594.3
	**Q3**	576.1	611.2	609.0	592.2	577.5	602.4	603.2	600.6	596.2	589.7	589.4	596.5	611.1	553.3	619.2	619.9	615.3	623.5
	**Max**	586.9	626.3	621.1	610.6	593.4	610.0	604.8	608.6	610.3	625.4	614.0	626.1	643.3	585.0	640.0	638.8	628.6	646.7
**Maui**	**Min**	410.9	395.5	376.7	384.0	392.3	407.7	399.0	421.0	383.5	366.5	391.8	388.4	392.2	346.9	424.6	402.1	446.5	442.5
	**Q1**	504.7	499.0	491.5	479.1	474.4	500.3	497.3	501.9	498.1	498.3	500.5	501.8	536.2	476.0	531.8	531.4	545.9	531.6
	**Q2**	540.6	543.7	536.4	531.0	509.7	541.7	526.7	543.5	530.4	543.5	538.8	536.0	572.9	511.6	570.2	557.2	565.0	550.2
	**Q3**	580.2	593.7	584.0	568.1	552.3	585.2	570.9	592.0	563.3	571.8	571.1	563.8	604.1	540.0	597.1	581.5	586.1	580.9
	**Max**	704.5	718.8	707.2	692.7	698.7	721.6	677.2	712.5	712.4	749.6	731.7	731.7	784.9	715.1	773.2	739.2	748.6	734.7
**Hawaii**	**Min**	377.4	347.9	348.7	380.5	354.8	398.4	401.9	391.1	360.6	367.1	381.7	356.2	381.1	333.4	387.8	400.1	414.6	399.9
	**Q1**	465.1	457.3	457.7	458.5	433.6	479.2	466.7	465.8	451.4	459.5	455.7	440.4	481.3	418.8	475.9	484.5	479.5	473.9
	**Q2**	526.6	527.2	526.4	520.8	501.4	536.0	519.9	529.8	513.4	526.8	517.7	501.6	560.6	489.3	539.3	543.2	532.6	528.2
	**Q3**	615.4	621.4	609.5	598.3	583.7	615.5	572.2	598.0	587.1	601.9	592.6	581.8	651.5	566.3	624.3	614.1	592.1	587.6
	**Max**	824.8	814.0	790.3	791.2	764.6	811.1	754.7	790.4	771.4	797.0	768.6	750.9	844.4	750.6	807.8	790.5	780.6	754.0

**Table 2:** The minimum values, the quartile values, and the maximum annual mean values for DHI across each island during each year from 1998 to 2015.

		**1998**	**1999**	**2000**	**2001**	**2002**	**2003**	**2004**	**2005**	**2006**	**2007**	**2008**	**2009**	**2010**	**2011**	**2012**	**2013**	**2014**	**2015**
**Niihau**	**Min**	144.4	152.9	146.6	154.5	157.6	151.7	155.9	146.1	153.9	145.4	149.4	153.0	149.6	158.5	145.8	141.7	141.5	126.8
	**Q1**	151.9	158.9	149.3	160.7	162.7	155.4	157.7	148.2	161.3	149.2	154.7	160.6	157.6	164.8	149.6	147.8	145.1	135.3
	**Q2**	157.8	161.1	151.4	163.1	165.9	157.0	159.8	152.4	163.3	152.8	160.6	164.0	163.1	166.8	152.6	152.2	148.4	138.8
	**Q3**	164.7	169.6	161.3	170.7	168.5	162.9	166.3	160.0	167.5	159.0	164.1	169.0	164.5	173.6	155.5	154.3	150.6	143.0
	**Max**	169.6	177.0	164.3	177.9	173.6	168.6	170.8	164.2	171.5	170.9	172.3	175.6	177.6	182.6	160.7	157.4	154.4	145.6
**Kauai**	**Min**	162.7	164.8	157.8	168.3	169.6	161.6	163.3	152.7	172.0	159.7	163.9	168.9	158.3	171.6	153.2	152.9	152.8	140.5
	**Q1**	181.4	186.1	177.0	187.7	188.1	177.3	183.5	173.5	183.3	178.9	177.4	186.3	177.1	187.6	170.4	168.2	169.5	160.6
	**Q2**	191.3	197.8	189.5	203.7	200.6	194.5	198.5	188.3	196.5	192.8	192.0	197.8	191.4	201.9	178.3	178.5	179.7	173.8
	**Q3**	200.6	211.4	205.7	218.8	213.9	207.3	208.1	205.0	209.0	212.9	209.5	215.1	209.1	217.1	195.6	197.0	197.8	192.2
	**Max**	214.3	226.0	221.3	231.8	228.3	226.1	225.5	221.9	220.0	229.2	220.8	224.3	220.9	232.1	213.1	213.2	216.3	208.1
**Oahu**	**Min**	159.8	149.9	146.4	152.9	158.5	147.9	150.3	141.7	153.5	143.5	154.4	154.7	149.2	163.8	147.6	142.6	143.7	133.2
	**Q1**	181.5	177.3	171.1	181.6	180.2	171.7	174.7	166.6	180.0	174.2	180.6	183.8	179.7	190.6	170.2	171.6	167.2	163.0
	**Q2**	192.1	194.7	191.4	199.9	198.2	188.8	193.7	185.4	192.8	188.4	189.7	195.5	187.2	201.2	181.4	181.4	178.0	172.7
	**Q3**	201.7	205.0	202.7	215.6	210.4	206.2	205.9	199.6	205.4	207.2	205.1	208.5	205.4	216.4	192.1	192.6	189.2	187.8
	**Max**	219.5	232.1	234.1	241.8	238.3	238.2	238.5	236.2	229.6	235.4	238.7	236.1	234.6	243.3	223.6	225.4	230.8	225.2
**Molokai**	**Min**	136.5	137.6	135.1	140.9	145.2	138.5	141.8	137.1	141.4	130.7	139.7	139.1	130.5	150.8	132.3	130.6	134.9	127.2
	**Q1**	149.2	146.5	147.7	152.5	158.5	149.3	152.5	146.9	162.0	154.7	159.1	161.3	148.1	169.1	146.1	151.9	152.4	145.3
	**Q2**	169.0	166.4	168.0	168.0	177.2	165.7	166.2	164.9	169.6	168.0	173.3	174.0	162.3	185.7	156.5	161.7	157.9	152.1
	**Q3**	182.1	186.9	187.0	190.0	195.1	186.5	187.8	180.5	181.6	175.5	182.5	180.8	174.5	192.3	167.7	170.5	166.2	165.7
	**Max**	196.6	209.7	211.5	224.3	215.1	211.4	209.2	208.4	206.0	217.1	213.7	218.7	211.8	227.5	191.1	199.5	188.6	189.5
**Lanai**	**Min**	132.4	133.8	133.6	137.5	143.3	138.3	140.5	136.0	143.4	127.0	133.2	137.1	119.7	145.7	129.8	130.1	133.8	123.1
	**Q1**	143.5	141.8	143.8	148.1	155.4	144.8	148.5	145.3	150.6	150.8	160.2	156.1	149.0	170.2	147.3	148.8	149.8	143.3
	**Q2**	159.5	161.9	160.3	163.5	174.8	167.0	170.6	165.4	167.6	162.8	167.1	166.5	155.2	178.4	159.6	161.8	157.0	152.7
	**Q3**	166.5	173.4	173.2	173.5	186.2	176.2	178.8	176.0	179.5	185.6	186.3	186.4	176.3	194.9	166.5	168.5	162.0	159.2
	**Max**	182.1	190.2	192.9	192.4	202.4	192.6	199.5	190.6	195.5	196.1	197.1	198.3	186.3	204.9	182.3	189.5	178.9	182.7
**Kahoolawe**	**Min**	162.3	144.1	145.8	149.0	159.3	150.8	150.9	150.2	149.8	143.7	147.1	144.7	138.1	159.6	138.0	138.2	143.8	138.2
	**Q1**	164.2	148.6	149.5	153.7	162.3	153.2	152.1	152.5	153.1	145.9	149.3	149.0	140.5	161.8	139.6	140.1	145.2	142.9
	**Q2**	165.1	150.5	152.2	158.0	164.6	155.3	152.9	153.7	154.5	158.2	156.6	154.7	154.0	171.8	145.0	144.7	148.2	147.5
	**Q3**	171.6	152.7	153.1	164.8	171.7	161.2	155.2	155.1	167.4	164.7	166.6	167.1	160.6	180.4	152.5	157.8	160.1	156.7
	**Max**	183.6	170.0	170.8	181.8	183.7	174.5	170.7	173.0	180.8	183.8	181.8	185.4	179.5	199.6	165.0	172.0	169.2	167.3
**Maui**	**Min**	139.5	132.8	135.7	142.3	139.7	130.8	149.0	135.3	134.7	117.0	123.6	124.4	100.2	129.1	104.0	117.9	116.8	120.7
	**Q1**	165.7	159.3	163.9	168.5	174.5	161.9	166.7	159.5	167.5	165.1	164.4	169.3	153.7	177.5	155.4	163.5	163.0	163.9
	**Q2**	177.9	176.8	182.3	188.4	192.2	182.1	184.1	179.6	182.9	177.5	179.3	184.1	167.5	190.9	166.9	172.0	170.9	175.0
	**Q3**	188.4	190.1	196.0	199.2	201.5	192.6	195.2	193.7	192.0	193.3	191.8	195.3	183.8	201.4	178.8	181.3	177.6	182.4
	**Max**	206.4	207.2	207.9	212.6	213.4	214.5	217.4	207.9	214.8	227.1	222.6	222.5	222.3	233.5	197.0	203.2	208.3	199.7
**Hawaii**	**Min**	94.6	99.6	110.7	107.3	122.8	101.7	117.1	107.3	115.3	103.8	117.4	124.4	82.3	122.8	96.2	103.7	110.4	122.3
	**Q1**	155.8	154.3	161.5	163.7	172.2	159.4	172.8	163.0	166.1	162.5	165.7	170.5	147.1	176.2	153.8	160.5	168.5	169.2
	**Q2**	175.2	176.1	183.8	185.7	193.6	183.8	188.1	183.3	185.9	181.8	182.7	188.6	171.4	193.7	172.3	177.8	183.5	182.8
	**Q3**	194.9	195.4	201.7	201.8	207.0	198.2	200.0	197.9	199.4	198.1	197.9	205.7	193.6	210.3	192.5	191.3	196.3	195.4
	**Max**	218.1	224.5	232.5	227.0	233.6	224.1	220.1	222.1	223.4	229.5	223.7	225.7	228.4	236.2	218.6	216.0	221.9	214.8

**Table 3:** The minimum values, the quartile values, and the maximum annual mean values for GHI across each island Hawaii during each year from 1998 to 2015.

		**1998**	**1999**	**2000**	**2001**	**2002**	**2003**	**2004**	**2005**	**2006**	**2007**	**2008**	**2009**	**2010**	**2011**	**2012**	**2013**	**2014**	**2015**
**Niihau**	**Min**	479.5	468.1	490.6	483.8	494.3	494.3	494.8	499.9	487.8	498.9	489.7	494.9	478.3	487.0	490.0	509.5	501.0	513.0
	**Q1**	494.2	487.8	504.2	493.1	497.3	503.2	502.7	514.4	491.8	510.2	495.1	496.6	493.4	495.6	492.1	510.1	504.6	514.5
	**Q2**	508.7	500.2	513.5	504.1	503.7	511.3	510.4	520.8	499.8	517.0	499.8	499.8	498.1	500.2	510.8	516.3	511.9	522.3
	**Q3**	527.5	521.8	535.4	524.9	513.1	520.6	521.0	536.8	507.0	529.2	514.2	522.1	517.0	518.5	524.2	528.1	523.0	535.1
	**Max**	541.7	525.7	540.6	531.9	524.9	530.8	527.7	541.8	520.6	538.5	532.4	534.3	532.8	525.9	531.4	537.8	532.2	554.5
**Kauai**	**Min**	322.0	313.9	342.7	344.1	348.9	359.0	346.8	362.7	352.5	354.6	364.1	358.8	348.7	350.3	284.3	318.2	321.4	332.3
	**Q1**	402.6	397.8	434.4	419.0	419.3	424.6	417.9	438.5	413.1	431.2	436.1	417.4	424.2	428.0	418.7	427.6	418.2	423.7
	**Q2**	439.8	436.3	460.5	448.0	445.0	454.2	443.7	461.4	441.1	461.5	458.2	455.5	462.1	453.8	461.7	462.8	456.3	467.2
	**Q3**	473.8	470.4	490.4	481.2	472.1	485.4	478.9	499.0	469.4	491.7	490.1	479.5	484.1	479.7	487.4	492.0	486.7	497.6
	**Max**	514.3	512.6	528.5	512.3	503.6	518.9	511.4	526.9	502.3	522.6	524.2	511.2	521.7	508.5	527.1	522.5	518.3	533.2
**Oahu**	**Min**	348.4	334.7	350.7	340.4	363.0	372.1	356.7	354.1	339.2	336.5	360.6	339.4	334.9	347.5	311.6	321.5	343.9	341.6
	**Q1**	423.0	423.4	441.3	433.5	437.7	449.9	438.9	452.4	418.9	431.4	433.3	423.9	425.0	430.0	426.8	434.8	441.0	430.2
	**Q2**	449.8	453.6	469.0	460.7	463.8	471.6	465.4	477.0	454.5	474.0	470.4	466.9	467.3	466.6	469.4	469.7	474.7	470.8
	**Q3**	484.5	489.0	503.4	494.9	495.3	501.6	497.9	510.6	480.5	491.3	490.7	485.6	489.3	488.6	493.3	488.9	492.6	493.7
	**Max**	530.8	535.6	547.6	539.5	532.4	543.5	535.0	553.1	524.6	546.4	536.9	533.8	535.6	531.7	533.7	534.8	537.5	542.5
**Molokai**	**Min**	379.0	382.2	381.8	377.1	400.9	409.6	408.5	397.1	382.1	366.9	399.3	385.0	366.9	378.7	361.8	379.1	409.5	386.1
	**Q1**	446.1	459.2	472.4	468.5	464.6	474.3	460.6	476.8	460.1	483.5	483.0	465.6	473.8	476.1	468.3	468.9	478.1	474.7
	**Q2**	509.4	517.7	511.3	513.0	507.5	515.7	514.9	519.6	503.5	509.0	497.2	513.3	512.1	499.3	517.5	509.2	507.8	513.7
	**Q3**	539.6	537.8	543.1	540.9	533.0	539.3	533.5	544.6	518.8	528.3	524.0	523.3	533.3	520.3	529.8	524.3	524.4	533.6
	**Max**	568.4	568.1	571.8	564.0	558.3	561.9	552.4	567.0	552.5	569.4	560.9	560.7	568.9	556.4	565.3	564.0	549.3	557.8
**Lanai**	**Min**	438.2	418.5	435.2	444.8	434.8	453.7	428.3	446.0	443.5	437.0	433.7	449.2	447.6	441.7	432.1	431.7	450.2	452.0
	**Q1**	490.4	478.0	480.7	482.8	481.9	489.7	476.8	483.0	472.6	459.9	457.2	463.7	464.6	459.6	478.4	471.8	492.6	494.7
	**Q2**	509.5	499.0	507.2	505.7	504.9	508.6	498.6	511.2	485.6	467.8	471.3	494.4	480.3	469.7	507.0	499.6	508.5	513.7
	**Q3**	554.0	555.3	552.9	544.1	543.2	548.1	542.6	549.4	521.1	528.4	522.4	516.0	536.7	519.9	538.4	520.3	516.8	525.3
	**Max**	575.7	569.7	568.3	562.0	556.1	563.4	552.8	563.0	550.1	577.8	571.0	564.0	588.1	563.8	575.2	564.8	558.8	569.9
**Kahoolawe**	**Min**	464.0	489.0	498.2	481.3	482.2	492.9	498.9	495.2	485.1	479.5	490.4	489.4	478.0	473.5	474.2	494.8	493.7	481.4
	**Q1**	473.6	489.8	498.4	487.3	484.8	496.6	499.7	495.7	492.9	488.6	502.6	501.1	487.0	485.2	477.1	497.2	498.2	485.6
	**Q2**	490.2	520.6	525.6	519.7	513.0	524.8	523.2	524.2	513.0	513.9	523.4	518.7	518.6	507.2	507.7	519.8	507.5	503.7
	**Q3**	517.3	539.3	547.3	528.3	523.6	527.7	532.7	536.3	518.1	524.7	530.8	527.9	524.3	514.8	522.8	531.3	524.1	518.7
	**Max**	521.4	545.5	550.9	546.0	536.2	539.9	537.0	541.7	530.5	543.3	544.9	548.3	549.6	534.0	539.0	544.1	537.9	542.7
**Maui**	**Min**	300.1	295.8	297.4	304.3	331.7	333.9	323.3	321.4	328.5	328.9	348.5	342.8	326.2	339.9	269.8	295.9	318.8	315.1
	**Q1**	416.7	430.8	434.8	432.5	428.8	443.7	432.6	433.1	426.8	433.7	437.7	429.9	443.3	434.0	430.6	431.2	440.1	425.1
	**Q2**	455.6	479.5	477.9	472.7	466.8	483.6	467.5	479.2	464.5	478.9	482.9	477.9	491.1	478.9	481.7	478.0	483.3	476.9
	**Q3**	511.0	523.2	522.8	511.8	503.3	519.8	509.6	525.8	506.0	521.1	521.4	517.0	532.0	518.5	526.2	513.7	511.6	511.6
	**Max**	552.0	567.9	564.3	553.5	562.7	569.0	539.2	558.3	554.5	583.1	576.5	578.2	606.1	576.0	599.6	580.3	577.8	579.1
**Hawaii**	**Min**	266.4	245.3	273.2	277.5	272.4	295.4	278.3	273.5	277.6	267.2	267.6	271.6	263.5	262.0	246.9	266.5	282.7	276.2
	**Q1**	376.0	380.3	397.3	399.5	393.0	412.3	407.6	403.7	387.2	397.8	402.0	384.0	401.3	390.4	381.8	393.9	397.8	390.3
	**Q2**	441.5	448.5	462.1	457.5	452.4	467.2	450.5	459.3	440.8	454.9	451.6	443.0	465.4	444.7	445.1	448.3	450.1	445.0
	**Q3**	503.0	503.0	510.2	505.1	495.8	510.5	493.5	506.0	490.0	500.9	497.4	490.1	523.2	493.0	504.3	490.8	486.5	483.6
	**Max**	634.9	617.5	612.9	612.6	591.4	623.5	594.2	608.7	589.4	615.0	590.2	581.4	648.9	587.5	625.5	608.0	597.8	587.2

**Table 4:** The minimum values, the quartile values, and the maximum annual mean values for dry bulb temperature (°C) across each island during each year from 1998 to 2015.

	**Year**	**1998**	**1999**	**2000**	**2001**	**2002**	**2003**	**2004**	**2005**	**2006**	**2007**	**2008**	**2009**	**2010**	**2011**	**2012**	**2013**	**2014**	**2015**
**Niihau**	**Min**	24.47	24.66	24.90	25.17	25.14	25.39	25.59	25.27	25.17	25.29	25.28	24.86	24.80	24.97	24.66	24.99	25.33	25.44
	**Q1**	24.51	24.70	24.94	25.20	25.18	25.42	25.62	25.30	25.20	25.33	25.32	24.90	24.84	25.00	24.70	25.03	25.37	25.48
	**Q2**	24.54	24.73	24.98	25.23	25.21	25.46	25.64	25.33	25.23	25.36	25.36	24.94	24.87	25.03	24.74	25.07	25.40	25.51
	**Q3**	24.59	24.78	25.04	25.27	25.25	25.51	25.67	25.37	25.27	25.41	25.42	24.99	24.92	25.07	24.80	25.13	25.45	25.56
	**Max**	24.65	24.83	25.10	25.32	25.30	25.57	25.72	25.42	25.32	25.47	25.48	25.05	24.98	25.11	24.86	25.19	25.50	25.62
**Kauai**	**Min**	24.39	24.55	24.80	25.05	25.05	25.34	25.46	25.16	25.05	25.20	25.21	24.82	24.76	24.89	24.64	24.99	25.30	25.40
	**Q1**	24.55	24.69	24.96	25.20	25.20	25.50	25.60	25.32	25.21	25.36	25.38	24.98	24.91	25.01	24.78	25.14	25.44	25.56
	**Q2**	24.67	24.79	25.06	25.32	25.34	25.65	25.72	25.46	25.33	25.48	25.50	25.13	25.05	25.11	24.88	25.26	25.58	25.71
	**Q3**	24.75	24.84	25.12	25.38	25.41	25.74	25.77	25.52	25.39	25.55	25.57	25.22	25.14	25.15	24.97	25.36	25.66	25.81
	**Max**	24.88	24.95	25.24	25.49	25.54	25.88	25.86	25.64	25.50	25.67	25.71	25.36	25.28	25.24	25.10	25.50	25.80	25.96
**Oahu**	**Min**	24.67	24.66	24.94	25.26	25.38	25.70	25.84	25.51	25.30	25.47	25.38	25.19	25.14	25.21	24.95	25.37	25.71	25.88
	**Q1**	24.91	24.85	25.16	25.47	25.61	25.94	26.04	25.74	25.50	25.68	25.63	25.45	25.39	25.40	25.19	25.63	25.95	26.16
	**Q2**	24.98	24.92	25.24	25.55	25.68	26.03	26.10	25.81	25.57	25.75	25.71	25.53	25.47	25.46	25.27	25.70	26.03	26.23
	**Q3**	25.09	25.01	25.34	25.65	25.79	26.14	26.19	25.91	25.65	25.85	25.82	25.64	25.58	25.55	25.39	25.82	26.14	26.34
	**Max**	25.19	25.09	25.43	25.73	25.89	26.25	26.26	26.01	25.74	25.94	25.92	25.75	25.69	25.62	25.50	25.92	26.25	26.45
**Molokai**	**Min**	24.66	24.59	24.87	25.14	25.32	25.60	25.88	25.49	25.23	25.40	25.25	25.14	25.08	25.18	24.84	25.35	25.67	25.92
	**Q1**	24.76	24.66	24.95	25.20	25.40	25.69	25.94	25.58	25.28	25.47	25.32	25.23	25.18	25.24	24.93	25.44	25.76	26.02
	**Q2**	24.82	24.71	25.01	25.25	25.45	25.76	25.99	25.65	25.33	25.52	25.37	25.29	25.25	25.28	24.98	25.50	25.83	26.10
	**Q3**	24.92	24.78	25.08	25.31	25.53	25.84	26.06	25.72	25.39	25.60	25.45	25.37	25.33	25.34	25.05	25.58	25.91	26.19
	**Max**	25.14	24.96	25.28	25.47	25.72	26.06	26.21	25.93	25.54	25.77	25.62	25.58	25.54	25.49	25.24	25.79	26.11	26.41
**Lanai**	**Min**	24.97	24.82	25.13	25.37	25.61	25.87	26.12	25.79	25.48	25.65	25.49	25.45	25.36	25.38	25.06	25.60	25.93	26.25
	**Q1**	25.00	24.86	25.17	25.40	25.65	25.92	26.15	25.83	25.51	25.69	25.53	25.49	25.41	25.41	25.10	25.65	25.98	26.29
	**Q2**	25.02	24.88	25.18	25.42	25.66	25.94	26.17	25.84	25.52	25.71	25.55	25.50	25.42	25.44	25.13	25.67	25.99	26.31
	**Q3**	25.07	24.90	25.21	25.44	25.69	25.98	26.19	25.88	25.53	25.73	25.57	25.54	25.46	25.45	25.15	25.70	26.03	26.35
	**Max**	25.10	24.94	25.25	25.47	25.72	26.02	26.21	25.91	25.56	25.76	25.61	25.57	25.50	25.48	25.20	25.75	26.07	26.39
**Kahoolawe**	**Min**	24.90	24.69	25.01	25.24	25.53	25.76	26.07	25.70	25.41	25.55	25.37	25.37	25.25	25.26	24.91	25.49	25.85	26.20
	**Q1**	24.90	24.70	25.01	25.25	25.54	25.77	26.07	25.70	25.41	25.55	25.37	25.37	25.26	25.26	24.91	25.49	25.85	26.20
	**Q2**	24.90	24.70	25.01	25.25	25.54	25.77	26.08	25.70	25.41	25.55	25.37	25.38	25.26	25.27	24.91	25.50	25.86	26.21
	**Q3**	24.90	24.70	25.01	25.25	25.54	25.77	26.08	25.70	25.42	25.55	25.38	25.38	25.26	25.27	24.91	25.50	25.86	26.21
	**Max**	24.90	24.70	25.02	25.25	25.54	25.77	26.08	25.70	25.42	25.55	25.38	25.38	25.26	25.27	24.92	25.50	25.86	26.21
**Maui**	**Min**	24.52	24.27	24.59	24.78	25.02	25.33	25.65	25.31	24.94	25.10	24.85	24.90	24.81	24.82	24.50	25.10	25.48	25.78
	**Q1**	24.65	24.41	24.73	24.91	25.18	25.48	25.78	25.44	25.07	25.23	25.01	25.04	24.95	24.96	24.64	25.23	25.61	25.92
	**Q2**	24.77	24.54	24.86	25.05	25.33	25.61	25.90	25.56	25.21	25.37	25.16	25.18	25.09	25.09	24.78	25.36	25.73	26.05
	**Q3**	24.96	24.75	25.07	25.26	25.54	25.84	26.07	25.75	25.38	25.57	25.40	25.40	25.32	25.29	25.01	25.58	25.93	26.25
	**Max**	25.28	25.09	25.42	25.58	25.86	26.21	26.32	26.07	25.64	25.90	25.76	25.73	25.68	25.60	25.38	25.93	26.25	26.56
**Hawaii**	**Min**	18.66	18.28	18.68	18.86	19.25	19.47	19.78	19.54	19.32	19.11	18.87	19.24	18.97	18.96	18.78	19.47	20.04	20.31
	**Q1**	21.40	21.03	21.45	21.61	21.96	22.18	22.47	22.23	21.91	21.82	21.53	21.84	21.69	21.63	21.41	22.10	22.63	22.94
	**Q2**	22.55	22.19	22.62	22.77	23.11	23.42	23.63	23.39	23.02	23.01	22.76	23.00	22.95	22.86	22.62	23.37	23.78	24.08
	**Q3**	24.06	23.76	24.18	24.34	24.67	24.89	25.09	24.88	24.58	24.51	24.29	24.53	24.43	24.39	24.15	24.79	25.22	25.53
	**Max**	25.47	25.05	25.47	25.62	25.90	26.08	26.22	26.08	25.81	25.67	25.53	25.81	25.71	25.59	25.37	25.91	26.42	26.87

The grand mean values of GHI and temperature were calculated among all NSRDB cells on both a monthly and annual basis. Additionally, the monthly grand means were compared on an interannual basis. The 1st, and 3rd quartiles of the 18 years of monthly grand means were calculated. On the same interannual basis, the mean, the standard deviation, the maximum, the minimum, and range of monthly grand means were calculated. Finally, the interannual coefficient of variation (COV), which is the standard deviation divided by the mean and expressed as a percentage, was calculated (see Table 5, Table 6, Table 7, Table 8, Table 9, Table 10, Table 11, Table 12, Table 13, Table 14, Table 15, Table 16, Table 17, Table 18, Table 19, Table 20).

**Table 5:** Monthly averaged global horizontal irradiance (W/m^2^) during daylight hours across Hawai’i from 1998 to 2015 with annual means and interannual statistics.

**Month**	**1998**	**1999**	**2000**	**2001**	**2002**	**2003**	**2004**	**2005**	**2006**	**2007**	**2008**	**2009**	**2010**	**2011**	**2012**	**2013**	**2014**	**2015**	**Q1**	**Mean**	**Q3**	**Sdev**	**COV**	**Min**	**Max**	**Range**
**January**	443.5	393.7	407.7	464.2	393.0	447.5	410.4	414.4	411.0	402.4	417.8	408.5	443.4	401.0	429.5	414.8	400.8	447.5	403.7	419.5	440.0	21.2	5.05%	393.0	464.2	71.1
**February**	490.9	426.8	515.6	416.7	459.8	465.6	445.8	444.2	416.8	433.7	431.7	426.9	476.0	433.5	452.2	456.5	420.4	487.9	428.1	450.1	464.1	28.2	6.27%	416.7	515.6	98.9
**March**	478.1	443.6	465.4	442.7	462.7	494.3	399.9	449.2	352.4	438.1	455.8	409.4	462.4	467.7	444.4	412.7	417.3	417.0	417.1	439.6	462.6	33.6	7.65%	352.4	494.3	141.9
**April**	453.3	473.6	489.9	462.4	472.5	451.4	446.2	493.9	463.4	499.2	465.1	427.1	485.0	453.1	455.4	488.4	487.0	472.2	453.8	468.8	486.5	19.3	4.12%	427.1	499.2	72.1
**May**	429.2	462.0	494.1	453.7	434.2	539.0	460.2	505.3	425.7	498.4	467.6	454.3	486.8	443.2	483.8	435.0	449.7	439.1	440.1	464.5	486.1	30.9	6.65%	425.7	539.0	113.2
**June**	438.6	479.1	462.2	463.3	472.1	460.4	463.7	454.4	479.2	475.7	464.3	451.9	491.1	464.4	452.1	485.5	474.0	443.1	455.9	465.3	475.3	14.2	3.04%	438.6	491.1	52.5
**July**	493.6	504.5	454.5	484.4	481.4	484.8	500.9	489.3	510.1	511.4	469.2	464.6	486.5	484.8	458.6	468.9	478.3	466.7	469.0	482.9	492.5	17.0	3.52%	454.5	511.4	56.9
**August**	470.8	475.6	463.1	510.9	454.7	502.5	516.1	484.0	491.4	479.4	517.1	483.1	505.8	486.6	471.8	499.3	496.2	434.5	472.7	485.7	501.7	22.0	4.52%	434.5	517.1	82.5
**September**	431.4	448.3	449.8	479.3	430.4	476.2	504.6	456.3	462.3	441.8	459.9	469.3	495.5	458.0	450.0	489.2	466.6	432.3	448.7	461.2	474.4	21.7	4.70%	430.4	504.6	74.2
**October**	431.1	433.3	429.7	432.1	432.8	455.9	420.3	422.3	406.9	448.5	428.5	435.8	422.1	432.8	448.1	408.9	426.6	434.8	423.4	430.6	434.4	12.4	2.89%	406.9	455.9	49.0
**November**	374.5	396.0	418.5	419.7	449.9	391.9	399.4	416.5	422.5	401.6	403.5	399.2	409.7	375.3	403.3	381.9	386.9	392.4	392.0	402.4	414.8	18.7	4.64%	374.5	449.9	75.4
**December**	381.2	395.9	441.2	412.6	439.6	398.7	409.6	449.8	432.5	360.4	372.5	445.2	397.3	382.0	397.3	385.9	421.3	428.5	388.4	408.4	431.5	26.9	6.59%	360.4	449.8	89.4
**Annual Mean**	**444.9**	**447.8**	**458.8**	**455.7**	**449.7**	**466.7**	**450.9**	**458.7**	**442.4**	**453.2**	**449.2**	**441.3**	**465.9**	**443.0**	**447.6**	**446.7**	**446.6**	**441.9**	**445.3**	**450.6**	**455.1**	**7.8**	**1.73%**	**441.3**	**466.7**	**25.4**

**Table 6:** Monthly averaged global horizontal irradiance (W/m^2^) during daylight hours across Kaho׳olawe from 1998 to 2015 with annual means and interannual statistics.

**Month**	**1998**	**1999**	**2000**	**2001**	**2002**	**2003**	**2004**	**2005**	**2006**	**2007**	**2008**	**2009**	**2010**	**2011**	**2012**	**2013**	**2014**	**2015**	**Q1**	**Mean**	**Q3**	**Sdev**	**COV**	**Min**	**Max**	**Range**
**January**	449.6	430.5	441.1	481.7	416.3	446.8	432.8	450.9	457.7	441.3	442.8	444.0	461.8	454.2	461.9	442.8	455.7	485.4	441.6	449.9	457.2	16.8	3.72%	416.3	485.4	69.2
**February**	527.1	531.3	544.9	509.4	517.0	535.1	486.8	506.8	500.1	517.9	509.6	498.2	510.1	504.0	523.6	474.3	494.0	510.0	501.1	511.1	522.2	17.5	3.43%	474.3	544.9	70.6
**March**	557.2	536.7	534.9	544.5	567.0	544.4	489.4	506.4	428.5	534.9	549.8	493.4	524.9	533.3	504.2	500.4	521.8	516.6	504.7	521.6	542.5	32.0	6.13%	428.5	567.0	138.5
**April**	542.7	587.1	578.1	571.9	564.1	562.7	586.1	589.6	556.1	590.5	573.9	539.5	570.4	598.7	566.0	600.0	563.6	556.9	562.9	572.1	586.8	17.6	3.08%	539.5	600.0	60.5
**May**	505.8	621.1	594.7	563.8	549.3	601.9	562.7	591.4	558.0	603.1	589.3	584.3	602.4	563.5	574.9	555.8	580.9	538.1	559.2	574.5	593.9	27.9	4.86%	505.8	621.1	115.3
**June**	532.2	562.7	579.3	537.1	555.4	526.2	567.9	580.7	585.2	560.7	559.7	604.6	562.8	540.1	511.7	547.0	553.2	536.1	537.9	555.7	566.6	23.2	4.17%	511.7	604.6	92.9
**July**	535.0	537.5	565.8	541.2	499.5	506.2	590.3	530.4	538.9	504.6	566.1	550.9	522.4	498.6	504.1	577.8	549.2	546.6	510.3	537.0	550.5	27.5	5.12%	498.6	590.3	91.7
**August**	544.6	542.3	537.9	543.4	513.1	546.3	560.0	581.0	550.2	560.9	551.6	535.1	520.9	511.1	550.1	599.0	565.3	515.2	535.8	546.0	557.9	23.0	4.21%	511.1	599.0	87.9
**September**	508.8	575.0	551.5	510.6	538.0	567.2	535.3	518.7	541.3	508.3	534.3	549.7	522.7	549.7	528.6	548.2	540.6	524.1	523.0	536.3	549.3	19.0	3.54%	508.3	575.0	66.7
**October**	445.8	471.6	468.5	484.4	480.4	492.9	493.9	476.0	486.3	468.4	476.5	507.5	483.6	479.3	500.3	496.7	459.7	498.7	472.7	481.7	493.7	15.6	3.24%	445.8	507.5	61.7
**November**	389.5	413.7	463.9	449.5	452.2	467.7	468.1	450.6	462.0	467.0	448.7	416.1	452.3	418.5	450.4	428.2	449.5	433.9	429.6	443.4	459.6	22.2	5.00%	389.5	468.1	78.6
**December**	433.0	420.2	471.2	442.6	479.9	418.8	447.8	462.5	442.1	386.5	425.1	495.6	441.7	419.2	423.7	449.8	423.0	449.2	423.2	440.7	449.6	25.9	5.87%	386.5	495.6	109.1
**Annual Mean**	**500.8**	**523.5**	**530.9**	**517.8**	**513.3**	**520.9**	**522.8**	**524.0**	**512.4**	**516.2**	**523.4**	**522.1**	**517.7**	**508.7**	**511.1**	**522.9**	**516.9**	**511.5**	**512.6**	**517.6**	**522.9**	**7.1**	**1.38%**	**500.8**	**530.9**	**30.2**

**Table 7:** Monthly averaged global horizontal irradiance (W/m^2^) during daylight hours across Kauai from 1998 to 2015 with annual means and interannual statistics.

**Month**	**1998**	**1999**	**2000**	**2001**	**2002**	**2003**	**2004**	**2005**	**2006**	**2007**	**2008**	**2009**	**2010**	**2011**	**2012**	**2013**	**2014**	**2015**	**Q1**	**Mean**	**Q3**	**Sdev**	**COV (%)**	**Min**	**Max**	**Range**
**January**	415.3	372.4	369.4	422.7	364.6	392.6	409.0	374.3	360.6	370.6	372.8	385.1	399.2	405.5	396.2	383.2	388.1	414.4	372.5	388.7	403.9	19.2	4.93	360.6	422.7	62.1
**February**	451.4	410.8	459.8	384.6	440.1	444.5	421.2	424.6	371.7	430.2	443.3	408.4	462.4	389.1	404.3	454.1	373.9	468.7	405.4	424.6	449.7	31.0	7.30	371.7	468.7	96.9
**March**	467.0	449.7	483.6	469.7	432.6	484.1	431.0	455.1	359.2	455.5	481.4	437.8	438.1	462.8	447.7	432.2	445.7	436.3	436.7	448.3	466.0	28.6	6.37	359.2	484.1	124.9
**April**	403.5	471.6	452.8	447.4	485.8	454.9	430.6	486.1	438.5	493.0	473.2	424.8	441.8	460.8	442.5	457.1	471.6	477.3	442.0	456.3	472.8	23.7	5.20	403.5	493.0	89.5
**May**	418.0	454.0	519.1	484.4	434.8	487.5	475.0	518.0	470.9	513.4	502.0	488.4	482.6	456.3	501.9	480.5	453.7	481.1	459.9	479.0	498.5	27.8	5.80	418.0	519.1	101.1
**June**	446.1	478.2	507.5	490.4	518.4	503.1	467.8	499.2	522.6	516.4	505.3	515.9	528.7	481.5	508.4	516.1	519.8	531.4	492.6	503.2	517.9	22.6	4.49	446.1	531.4	85.3
**July**	508.0	467.0	494.0	506.8	483.2	523.6	543.2	498.9	522.1	518.3	514.6	489.6	485.2	524.8	488.5	530.0	523.0	530.0	490.7	508.4	523.5	20.5	4.03	467.0	543.2	76.3
**August**	474.6	478.9	492.4	475.7	467.9	505.5	485.4	531.0	491.4	494.0	506.9	472.6	487.8	501.1	506.6	522.4	514.5	480.9	479.4	493.9	506.3	17.9	3.63	467.9	531.0	63.1
**September**	482.1	470.7	441.2	479.7	459.5	478.6	493.8	468.8	484.1	484.6	488.9	501.2	494.5	502.0	482.7	478.8	496.4	468.3	472.7	480.9	492.6	15.3	3.19	441.2	502.0	60.8
**October**	426.0	407.3	434.4	402.6	420.0	404.7	434.4	418.9	397.3	415.4	402.6	408.7	451.3	416.4	443.0	424.8	412.7	437.7	407.7	419.9	432.3	15.4	3.67	397.3	451.3	53.9
**November**	353.6	371.6	394.5	390.6	380.2	374.7	365.1	389.1	410.2	396.3	380.0	391.1	390.3	386.0	398.8	385.0	392.0	371.2	376.0	384.5	391.8	13.5	3.52	353.6	410.2	56.6
**December**	338.8	318.2	377.5	355.9	377.0	343.7	307.1	416.0	370.5	323.1	335.6	395.4	334.1	364.4	345.6	382.4	372.9	386.0	336.4	358.0	377.4	29.2	8.15	307.1	416.0	108.9
**Annual Mean**	**435.2**	**433.8**	**456.0**	**446.6**	**441.9**	**454.3**	**444.2**	**461.3**	**439.1**	**456.6**	**456.4**	**446.9**	**453.6**	**450.2**	**452.3**	**458.3**	**452.1**	**460.6**	**444.8**	**450.0**	**456.3**	**8.4**	**1.86**	**433.8**	**461.3**	**27.5**

**Table 8:** Monthly averaged global horizontal irradiance (W/m^2^) during daylight hours across Lanai from 1998 to 2015 with annual means and interannual statistics.

**Month**	**1998**	**1999**	**2000**	**2001**	**2002**	**2003**	**2004**	**2005**	**2006**	**2007**	**2008**	**2009**	**2010**	**2011**	**2012**	**2013**	**2014**	**2015**	**Q1**	**Mean**	**Q3**	**Sdev**	**COV (%)**	**Min**	**Max**	**Range**
**January**	445.8	426.0	449.6	447.4	417.1	419.9	418.4	408.6	438.7	414.0	437.9	429.5	431.3	431.1	417.8	411.6	433.2	450.8	417.9	429.4	438.5	13.6	3.16	408.6	450.8	42.2
**February**	519.5	502.3	505.4	495.5	526.6	537.6	451.1	507.5	479.7	496.0	471.5	493.9	498.4	441.9	483.4	527.7	441.6	478.6	478.9	492.1	507.0	28.1	5.72	441.6	537.6	96.0
**March**	542.0	528.5	518.6	500.4	534.6	504.7	465.9	518.5	397.8	481.6	504.1	481.8	542.2	520.0	510.0	475.3	514.5	509.1	486.5	502.8	519.7	34.1	6.79	397.8	542.2	144.3
**April**	569.9	554.7	561.6	553.0	531.3	560.1	562.9	567.4	562.7	584.4	525.7	513.8	543.2	510.6	551.0	546.3	567.7	576.5	544.0	552.4	566.3	20.7	3.74	510.6	584.4	73.8
**May**	535.6	519.8	540.9	535.9	502.2	575.7	536.3	556.7	549.2	520.3	534.3	501.1	504.8	504.6	580.2	505.3	488.7	548.4	505.0	530.0	546.5	26.2	4.94	488.7	580.2	91.6
**June**	528.1	563.8	563.5	545.2	557.8	563.4	557.3	558.0	565.1	563.8	521.9	550.5	544.2	517.1	540.3	546.0	584.2	582.4	544.4	552.9	563.7	18.4	3.32	517.1	584.2	67.0
**July**	585.6	557.9	546.0	570.8	545.2	554.3	576.5	559.5	555.9	531.7	554.4	527.0	544.3	541.0	543.0	552.6	566.8	561.8	544.5	554.1	561.2	15.0	2.70	527.0	585.6	58.6
**August**	572.0	570.1	566.6	558.4	531.6	572.7	550.8	566.8	553.8	527.0	557.6	563.1	552.3	539.3	566.0	578.3	583.4	526.9	551.1	557.6	569.3	17.0	3.05	526.9	583.4	56.6
**September**	576.7	571.2	529.1	564.8	545.1	544.9	565.2	552.4	526.3	508.5	533.2	555.5	549.3	545.5	521.2	547.4	535.0	535.3	533.7	544.8	554.7	18.0	3.30	508.5	576.7	68.1
**October**	480.4	476.9	479.2	493.1	478.4	478.9	473.5	477.2	434.1	482.1	441.7	467.0	481.1	460.6	476.6	468.1	466.3	486.1	467.3	472.3	480.1	14.7	3.11	434.1	493.1	59.0
**November**	424.8	443.5	459.5	443.7	452.4	454.5	435.4	415.6	438.6	443.7	415.5	449.6	433.0	439.5	449.4	415.6	450.7	437.8	433.6	439.0	449.5	13.6	3.11	415.5	459.5	44.0
**December**	411.5	390.3	448.8	426.7	471.6	421.7	439.3	465.4	436.1	390.2	386.5	466.4	406.6	437.5	421.1	439.5	434.0	443.9	413.9	429.8	442.8	25.7	5.98	386.5	471.6	85.2
**Annual Mean**	**520.3**	**513.3**	**517.4**	**515.0**	**510.2**	**519.6**	**507.8**	**517.2**	**499.6**	**499.7**	**495.4**	**502.6**	**506.3**	**494.1**	**509.6**	**504.9**	**510.3**	**515.3**	**503.2**	**508.8**	**515.3**	**8.1**	**1.60**	**494.1**	**520.3**	**26.1**

**Table 9:** Monthly averaged global horizontal irradiance (W/m^2^) during daylight hours across Maui from 1998 to 2015 with annual means and interannual statistics.

**Month**	**1998**	**1999**	**2000**	**2001**	**2002**	**2003**	**2004**	**2005**	**2006**	**2007**	**2008**	**2009**	**2010**	**2011**	**2012**	**2013**	**2014**	**2015**	**Q1**	**Mean**	**Q3**	**Sdev**	**COV (%)**	**Min**	**Max**	**Range**
**January**	410.0	386.3	394.0	444.7	384.7	407.3	400.1	405.1	403.8	397.6	395.3	411.3	418.8	412.3	421.5	392.3	406.2	438.4	395.8	407.2	412.0	16.1	3.96	384.7	444.7	60.0
**February**	476.1	440.3	502.6	445.4	457.4	489.1	428.8	463.8	412.4	447.6	446.9	429.1	448.2	429.8	452.8	473.1	426.1	468.1	432.4	452.1	467.0	23.5	5.21	412.4	502.6	90.2
**March**	487.6	470.2	472.3	451.4	464.7	483.6	405.7	452.5	373.0	442.6	483.0	452.4	467.9	490.8	449.2	431.3	442.0	448.8	444.2	453.8	471.8	29.6	6.51	373.0	490.8	117.9
**April**	440.3	512.4	498.5	489.7	486.1	495.7	472.7	501.3	479.1	525.3	497.6	445.2	524.6	504.2	502.4	501.2	501.0	506.8	487.0	493.6	503.8	22.7	4.61	440.3	525.3	85.0
**May**	450.8	510.3	528.0	482.5	476.2	542.0	495.3	532.6	458.4	525.3	522.2	476.2	515.4	503.0	530.2	460.8	484.6	469.4	476.2	498.0	524.5	29.0	5.82	450.8	542.0	91.2
**June**	470.6	532.3	519.1	489.4	502.6	515.4	518.0	507.5	536.2	527.1	536.0	521.7	536.3	504.9	496.5	515.6	538.6	511.4	505.5	515.5	531.0	18.3	3.55	470.6	538.6	68.0
**July**	538.9	529.9	491.1	530.3	500.2	509.3	546.2	514.5	535.7	525.7	526.9	504.6	522.7	524.3	497.5	515.9	526.5	528.9	510.6	520.5	529.6	15.1	2.89	491.1	546.2	55.1
**August**	507.5	497.5	485.6	513.9	477.9	516.1	526.1	520.7	532.4	517.6	542.4	519.9	540.6	504.8	527.0	551.2	545.7	469.4	505.4	516.5	531.0	23.1	4.47	469.4	551.2	81.8
**September**	480.8	500.4	462.2	498.0	472.8	504.7	516.5	487.9	504.0	471.8	494.0	527.3	521.8	510.5	488.3	534.5	519.7	474.4	482.6	498.3	515.0	21.0	4.21	462.2	534.5	72.4
**October**	435.3	444.5	434.0	432.6	449.7	459.2	440.5	440.5	428.7	457.0	442.1	455.1	453.5	445.1	476.2	437.5	440.0	443.3	438.1	445.3	452.5	11.6	2.60	428.7	476.2	47.5
**November**	379.4	397.9	423.6	422.0	421.4	397.7	399.9	399.0	422.8	424.0	406.2	420.7	418.0	404.4	420.2	387.0	397.5	406.4	398.2	408.2	421.3	13.8	3.39	379.4	424.0	44.5
**December**	373.9	377.6	433.2	390.8	440.6	362.5	393.5	437.6	421.9	371.0	386.7	452.2	400.8	396.9	395.7	418.1	409.7	432.4	387.7	405.3	429.7	26.7	6.58	362.5	452.2	89.7
**Annual Mean**	**458.3**	**471.8**	**473.2**	**469.1**	**463.5**	**478.0**	**467.0**	**475.5**	**463.8**	**474.5**	**478.8**	**471.1**	**485.1**	**473.3**	**475.4**	**471.8**	**474.5**	**468.9**	**469.0**	**471.9**	**475.2**	**6.2**	**1.32**	**458.3**	**485.1**	**26.8**

**Table 10:** Monthly averaged global horizontal irradiance (W/m^2^) during daylight hours across Moloka׳I from 1998 to 2015 with annual means and interannual statistics.

**Month**	**1998**	**1999**	**2000**	**2001**	**2002**	**2003**	**2004**	**2005**	**2006**	**2007**	**2008**	**2009**	**2010**	**2011**	**2012**	**2013**	**2014**	**2015**	**Q1**	**Mean**	**Q3**	**Sdev**	**COV (%)**	**Min**	**Max**	**Range**
**January**	443.4	396.4	411.4	437.1	396.7	402.1	412.8	390.1	411.6	411.4	414.5	407.2	416.6	428.9	413.2	385.8	414.0	433.8	403.4	412.6	416.1	15.7	3.79	385.8	443.4	57.6
**February**	504.4	475.9	505.9	469.7	472.4	499.9	437.5	480.1	439.4	474.9	470.3	445.1	466.0	448.0	461.1	485.2	421.5	455.2	449.8	467.4	479.0	23.5	5.03	421.5	505.9	84.4
**March**	518.2	498.3	508.9	498.7	497.0	488.3	437.3	484.0	379.2	483.9	514.6	472.6	499.3	510.6	489.0	462.1	483.0	484.9	483.2	483.9	499.2	32.6	6.74	379.2	518.2	139.0
**April**	503.0	559.1	517.2	527.8	523.2	558.0	539.1	542.3	524.0	561.2	512.4	497.5	516.9	521.9	525.5	547.3	524.6	571.9	518.4	531.8	546.1	21.0	3.94	497.5	571.9	74.4
**May**	508.0	524.6	549.0	522.3	523.1	568.2	526.9	573.4	533.8	539.6	546.1	523.4	530.3	518.9	570.5	514.7	504.5	536.1	522.5	534.1	544.5	20.5	3.83	504.5	573.4	68.9
**June**	521.6	570.2	570.6	538.4	553.4	559.6	558.4	559.1	578.2	572.8	551.2	551.6	556.0	517.8	534.1	545.1	580.7	585.2	546.6	555.8	570.5	19.2	3.46	517.8	585.2	67.4
**July**	555.5	553.6	536.5	573.7	527.8	541.6	589.5	559.6	548.9	544.1	556.3	534.9	529.1	541.0	545.2	539.6	568.4	581.3	539.9	551.5	558.8	17.5	3.18	527.8	589.5	61.7
**August**	552.5	535.2	529.7	540.3	529.1	557.7	554.5	561.2	551.5	547.0	560.0	543.2	556.3	540.1	562.7	586.7	575.4	513.6	540.2	549.8	559.4	17.4	3.16	513.6	586.7	73.2
**September**	540.7	550.4	523.6	538.2	528.2	537.1	556.5	525.4	531.5	516.6	519.7	554.6	552.1	551.0	535.1	547.0	557.3	527.6	527.8	538.5	550.8	13.3	2.47	516.6	557.3	40.7
**October**	461.4	464.0	462.8	461.1	472.0	478.8	460.6	455.7	435.5	464.1	440.5	464.6	472.6	452.8	476.3	463.3	462.3	448.8	456.9	460.9	464.5	11.3	2.45	435.5	478.8	43.3
**November**	392.0	417.9	445.2	436.9	436.8	419.2	427.3	413.5	438.4	438.8	414.7	427.7	435.5	420.8	440.3	404.4	426.6	406.7	415.5	424.6	436.9	14.6	3.43	392.0	445.2	53.2
**December**	399.1	373.7	436.4	391.4	451.0	382.9	405.2	456.5	430.9	371.1	381.7	450.8	404.3	401.9	387.0	427.7	404.1	420.5	388.1	409.8	430.1	27.2	6.64	371.1	456.5	85.4
**Annual Mean**	**495.9**	**499.0**	**503.8**	**499.1**	**496.0**	**504.9**	**498.2**	**505.3**	**489.4**	**499.5**	**496.2**	**493.6**	**499.2**	**492.1**	**500.6**	**497.5**	**499.7**	**502.3**	**496.1**	**498.5**	**500.3**	**4.2**	**0.85**	**489.4**	**505.3**	**15.9**

**Table 11:** Monthly averaged global horizontal irradiance (W/m^2^) during daylight hours across Ni׳ihau from 1998 to 2015 with annual means and interannual statistics.

**Month**	**1998**	**1999**	**2000**	**2001**	**2002**	**2003**	**2004**	**2005**	**2006**	**2007**	**2008**	**2009**	**2010**	**2011**	**2012**	**2013**	**2014**	**2015**	**Q1**	**Mean**	**Q3**	**Sdev**	**COV (%)**	**Min**	**Max**	**Range**
**January**	450.6	446.1	443.0	463.2	409.9	430.6	453.3	426.8	444.8	447.1	436.8	450.0	425.3	433.4	443.6	455.5	448.3	432.6	432.8	441.2	449.6	12.9	2.93	409.9	463.2	53.3
**February**	523.1	506.6	518.9	472.2	517.5	514.0	493.6	482.4	423.6	515.9	503.8	495.2	523.7	437.5	476.3	518.2	420.7	517.3	477.8	492.3	517.4	33.9	6.89	420.7	523.7	103.0
**March**	562.7	547.4	562.5	551.9	513.5	551.1	532.1	536.2	405.7	536.4	548.0	510.9	547.9	544.0	501.8	504.4	548.7	527.0	516.9	529.6	548.5	36.1	6.82	405.7	562.7	157.0
**April**	525.8	559.2	514.6	546.7	559.3	523.1	520.7	575.6	551.8	573.4	531.8	515.9	519.7	530.5	533.0	544.7	549.6	565.8	523.8	541.2	557.4	20.0	3.69	514.6	575.6	61.0
**May**	510.2	552.0	574.8	565.0	535.5	548.5	567.1	573.1	570.6	567.7	552.4	569.3	559.4	540.0	588.2	587.6	542.6	562.5	549.4	559.2	570.3	19.3	3.45	510.2	588.2	78.0
**June**	554.4	548.5	574.8	553.2	576.4	577.5	547.7	605.3	572.2	583.3	553.0	597.4	557.5	566.8	571.3	573.1	590.2	577.8	555.2	571.1	577.7	16.6	2.91	547.7	605.3	57.6
**July**	585.9	523.1	562.7	570.2	538.8	573.6	602.3	561.7	570.7	567.0	558.6	546.2	531.4	570.8	561.2	583.1	559.9	589.8	558.9	564.3	572.9	20.2	3.59	523.1	602.3	79.3
**August**	541.3	558.1	557.1	519.8	524.3	570.9	548.2	575.1	549.5	558.5	548.3	516.3	536.9	544.9	542.6	563.5	577.3	565.8	541.6	549.9	562.3	17.9	3.26	516.3	577.3	61.0
**September**	551.7	522.7	506.5	524.4	539.2	512.2	563.0	542.7	547.7	529.4	535.7	549.4	515.6	527.1	559.4	545.5	555.0	561.1	525.0	538.2	551.1	17.4	3.24	506.5	563.0	56.5
**October**	485.1	442.6	498.3	468.0	466.5	463.0	494.9	484.8	449.1	472.6	452.4	459.1	495.1	460.8	470.2	468.7	465.3	521.3	461.4	473.2	485.0	19.9	4.21	442.6	521.3	78.7
**November**	440.6	431.4	472.3	428.8	444.4	465.3	414.7	468.3	451.4	457.8	439.0	450.8	452.7	462.4	433.7	443.6	460.1	429.7	435.0	447.1	459.5	15.8	3.53	414.7	472.3	57.5
**December**	398.0	378.4	427.3	419.8	428.4	404.4	355.6	433.3	428.4	405.8	387.7	422.1	381.0	422.1	410.2	418.3	413.3	442.9	399.6	409.8	426.0	22.4	5.48	355.6	442.9	87.3
**Annual Mean**	**514.0**	**505.8**	**521.2**	**510.8**	**507.8**	**515.2**	**514.2**	**526.8**	**503.3**	**522.2**	**508.9**	**510.4**	**507.7**	**507.8**	**512.7**	**521.4**	**516.3**	**528.7**	**508.1**	**514.2**	**520.0**	**7.3**	**1.42**	**503.3**	**528.7**	**25.4**

**Table 12:** Monthly averaged global horizontal irradiance (W/m^2^) during daylight hours across O׳ahu from 1998 to 2015 with annual means and interannual statistics.

**Month**	**1998**	**1999**	**2000**	**2001**	**2002**	**2003**	**2004**	**2005**	**2006**	**2007**	**2008**	**2009**	**2010**	**2011**	**2012**	**2013**	**2014**	**2015**	**Q1**	**Mean**	**Q3**	**Sdev**	**COV (%)**	**Min**	**Max**	**Range**
**January**	419.9	368.3	391.0	394.1	376.3	378.6	391.0	359.7	366.7	376.5	379.8	370.3	401.4	406.5	390.2	358.8	400.1	398.5	371.8	384.9	397.4	17.0	4.42	358.8	419.9	61.0
**February**	449.7	451.1	466.9	422.4	444.4	455.8	419.4	440.4	386.5	435.8	445.4	415.3	434.0	425.0	417.4	441.6	394.8	457.2	420.2	433.5	448.6	21.5	4.95	386.5	466.9	80.5
**March**	477.0	456.4	485.2	456.6	472.2	467.2	441.0	469.6	339.6	439.7	491.5	447.6	447.8	454.3	440.3	450.1	459.1	456.2	447.7	452.9	469.0	32.0	7.07	339.6	491.5	151.9
**April**	445.8	502.1	451.8	476.1	498.5	515.9	496.3	504.4	470.3	498.7	484.5	453.9	466.0	483.1	459.8	460.4	475.4	508.4	461.8	480.6	498.6	21.6	4.49	445.8	515.9	70.0
**May**	452.9	464.5	505.0	495.7	468.2	529.6	490.5	521.2	495.2	499.8	504.2	486.6	468.0	468.2	522.1	485.6	456.2	492.3	468.2	489.2	503.1	22.7	4.65	452.9	529.6	76.7
**June**	463.9	504.3	545.0	493.3	530.5	509.3	494.1	521.1	537.5	543.6	510.5	514.2	501.6	482.2	502.3	485.0	542.3	538.1	496.0	512.2	535.7	23.8	4.65	463.9	545.0	81.1
**July**	496.7	479.3	496.9	518.3	502.2	528.8	547.3	527.7	516.5	508.2	531.7	493.9	492.7	505.8	498.8	504.9	529.8	536.9	497.4	512.0	528.5	18.4	3.60	479.3	547.3	68.0
**August**	504.2	514.4	510.5	491.7	483.0	515.3	486.1	538.3	507.4	513.0	513.0	489.8	497.1	499.0	519.5	544.4	527.0	482.6	493.1	507.6	515.1	17.9	3.53	482.6	544.4	61.9
**September**	501.0	513.0	466.9	516.6	492.4	505.6	517.0	483.9	499.6	500.2	499.3	512.3	520.0	522.5	479.5	493.6	521.6	452.1	492.7	499.8	515.7	19.5	3.90	452.1	522.5	70.3
**October**	451.8	417.5	438.6	420.2	435.7	431.0	443.5	430.3	395.3	435.5	416.8	437.5	442.1	429.5	440.6	422.6	435.9	423.7	422.9	430.5	438.4	13.0	3.02	395.3	451.8	56.5
**November**	359.6	378.9	397.9	398.0	408.8	392.5	394.0	385.0	417.0	403.2	379.9	393.1	400.0	401.2	406.6	396.4	418.8	366.9	386.9	394.3	402.7	15.6	3.97	359.6	418.8	59.2
**December**	346.2	336.7	394.4	356.5	411.0	354.1	370.7	428.9	384.2	342.5	335.1	411.1	370.8	377.2	341.2	402.9	380.0	388.8	348.2	374.0	393.0	28.5	7.63	335.1	428.9	93.9
**Annual Mean**	**451.3**	**453.8**	**466.7**	**457.6**	**463.5**	**470.4**	**462.7**	**472.8**	**449.2**	**463.8**	**463.8**	**456.3**	**457.3**	**458.1**	**456.8**	**458.4**	**466.8**	**463.1**	**457.0**	**460.7**	**463.8**	**6.4**	**1.38**	**449.2**	**472.8**	**23.6**

**Table 13:** Monthly averaged dry-bulb temperature (°C) across Hawai׳i for years 1998–2015 with annual means and interannual statistics of monthly values.

**Month**	**1998**	**1999**	**2000**	**2001**	**2002**	**2003**	**2004**	**2005**	**2006**	**2007**	**2008**	**2009**	**2010**	**2011**	**2012**	**2013**	**2014**	**2015**	**Q1**	**Mean**	**Q3**	**Sdev**	**COV (%)**	**Min**	**Max**	**Range**
**January**	22.3	21.4	20.4	21.9	21.7	22.2	22.5	22.8	21.7	21.7	20.8	21.2	22.8	22.0	21.8	21.5	22.2	22.5	21.5	21.9	22.3	0.6	2.96	20.4	22.8	2.4
**February**	21.5	21.0	21.5	21.2	20.9	21.2	22.7	21.7	21.2	21.3	21.7	20.1	21.8	22.6	21.5	20.5	23.2	23.2	21.2	21.6	21.7	0.8	3.91	20.1	23.2	3.0
**March**	22.4	21.0	21.6	21.4	21.7	22.9	22.3	22.2	21.9	22.4	21.9	20.6	21.3	22.2	21.3	21.8	22.6	22.0	21.5	21.9	22.3	0.6	2.72	20.6	22.9	2.3
**April**	21.1	21.2	21.0	21.5	23.0	22.2	22.8	22.4	21.7	22.2	22.2	21.6	21.8	22.9	21.7	23.2	22.4	22.8	21.6	22.1	22.7	0.7	3.10	21.0	23.2	2.1
**May**	21.5	22.3	22.6	22.6	23.6	23.3	23.9	23.8	22.5	23.0	22.6	24.0	22.8	22.8	22.4	24.0	23.9	23.1	22.6	23.0	23.7	0.7	3.05	21.5	24.0	2.4
**June**	22.8	22.6	23.5	23.0	23.8	24.1	24.0	23.7	23.6	23.7	23.4	24.0	23.4	23.0	22.9	23.7	23.9	24.6	23.1	23.5	23.9	0.5	2.19	22.6	24.6	2.0
**July**	23.5	23.2	23.8	23.9	24.1	24.3	24.5	24.2	24.1	24.0	24.2	24.2	23.7	23.5	23.5	24.4	24.7	25.9	23.8	24.1	24.3	0.6	2.50	23.2	25.9	2.7
**August**	24.3	23.5	24.2	24.4	24.5	24.6	25.0	24.8	24.7	24.5	24.0	24.8	23.8	23.9	23.9	24.7	25.2	26.3	24.0	24.5	24.8	0.6	2.59	23.5	26.3	2.8
**September**	23.8	23.4	24.4	24.3	24.3	25.0	25.1	24.8	24.3	24.3	24.2	24.6	24.0	24.1	23.8	24.7	25.8	26.2	24.1	24.5	24.8	0.7	2.83	23.4	26.2	2.8
**October**	23.4	23.3	23.5	23.8	24.1	25.0	24.5	23.6	24.6	23.6	23.6	24.9	23.7	23.5	24.1	24.6	25.1	25.6	23.6	24.1	24.6	0.7	2.80	23.3	25.6	2.3
**November**	22.4	21.9	22.5	23.4	23.4	23.1	23.8	23.5	24.0	23.0	22.8	23.1	23.0	22.3	22.7	23.6	23.8	24.0	22.7	23.1	23.6	0.6	2.61	21.9	24.0	2.1
**December**	21.4	21.6	22.2	22.1	22.2	22.4	22.3	22.8	22.2	22.0	21.7	22.9	22.5	21.4	21.9	22.7	22.4	22.7	21.9	22.2	22.5	0.5	2.04	21.4	22.9	1.5
**Annual Mean**	**22.6**	**22.2**	**22.6**	**22.8**	**23.1**	**23.4**	**23.6**	**23.4**	**23.1**	**23.0**	**22.8**	**23.0**	**22.9**	**22.8**	**22.6**	**23.3**	**23.8**	**24.1**	**22.8**	**23.1**	**23.4**	**0.5**	**2.02**	**22.2**	**24.1**	**1.9**

**Table 14:** Monthly averaged dry-bulb temperature (°C) across Kaho׳olawe for years 1998–2015 with annual means and interannual statistics of monthly values.

**Month**	**1998**	**1999**	**2000**	**2001**	**2002**	**2003**	**2004**	**2005**	**2006**	**2007**	**2008**	**2009**	**2010**	**2011**	**2012**	**2013**	**2014**	**2015**	**Q1**	**Mean**	**Q3**	**Sdev**	**COV (%)**	**Min**	**Max**	**Range**
**January**	24.4	23.7	23.0	24.5	24.1	24.3	24.7	24.8	24.4	24.6	23.7	23.8	24.7	24.4	24.3	23.7	24.2	24.3	23.9	24.2	24.5	0.5	1.88	23.0	24.8	1.8
**February**	23.9	23.5	23.9	24.0	23.3	23.7	25.1	24.2	23.4	23.7	24.0	23.0	24.1	25.0	23.9	23.1	24.9	24.8	23.5	24.0	24.2	0.6	2.62	23.0	25.1	2.1
**March**	24.6	23.5	23.9	24.0	24.0	25.0	24.4	24.0	24.2	24.7	24.5	22.8	23.8	24.5	23.5	23.6	24.3	24.1	23.8	24.1	24.5	0.5	2.16	22.8	25.0	2.2
**April**	23.6	23.8	23.3	24.0	25.3	24.9	24.9	24.7	23.9	24.7	24.6	23.5	24.2	25.4	24.1	25.0	24.5	25.3	23.9	24.4	24.9	0.7	2.69	23.3	25.4	2.1
**May**	23.8	25.0	24.8	24.9	25.9	25.6	26.0	26.0	24.5	25.5	25.2	25.9	25.3	25.3	24.6	25.8	26.1	25.1	24.9	25.3	25.9	0.6	2.49	23.8	26.1	2.3
**June**	25.0	25.0	25.8	25.3	26.2	26.3	26.4	26.1	26.0	26.3	26.0	26.4	25.6	25.3	25.0	25.8	26.2	26.4	25.4	25.8	26.3	0.5	1.97	25.0	26.4	1.4
**July**	25.6	25.4	26.1	26.3	26.3	26.6	27.2	26.6	26.3	26.4	26.8	26.7	25.9	25.7	25.5	26.6	26.9	27.9	26.0	26.4	26.7	0.6	2.35	25.4	27.9	2.4
**August**	26.4	26.0	26.5	26.6	26.9	27.0	27.7	27.2	27.0	26.9	26.5	27.0	26.1	26.1	26.0	26.8	27.3	28.4	26.4	26.8	27.0	0.6	2.29	26.0	28.4	2.4
**September**	26.3	25.8	26.7	26.7	26.9	27.5	27.7	27.2	26.8	26.9	26.6	26.9	26.3	26.3	26.0	27.1	28.0	28.4	26.4	26.9	27.1	0.7	2.51	25.8	28.4	2.5
**October**	25.9	25.8	26.0	26.2	26.5	27.5	27.1	26.3	26.9	26.4	26.3	27.4	26.3	26.1	26.2	26.9	27.4	27.7	26.2	26.6	27.0	0.6	2.21	25.8	27.7	1.9
**November**	25.1	24.6	25.1	25.7	26.0	25.7	26.5	25.9	26.4	25.8	25.5	25.7	25.6	24.9	25.1	26.0	26.0	26.6	25.2	25.7	26.0	0.6	2.17	24.6	26.6	2.0
**December**	24.1	24.1	24.9	24.6	25.0	24.9	25.1	25.2	25.0	24.7	24.7	25.2	25.1	24.0	24.6	25.5	24.5	25.5	24.6	24.8	25.1	0.4	1.79	24.0	25.5	1.5
**Annual Mean**	**24.9**	**24.7**	**25.0**	**25.2**	**25.5**	**25.8**	**26.1**	**25.7**	**25.4**	**25.6**	**25.4**	**25.4**	**25.3**	**25.3**	**24.9**	**25.5**	**25.9**	**26.2**	**25.3**	**25.4**	**25.7**	**0.4**	**1.59**	**24.7**	**26.2**	**1.5**

**Table 15:** Monthly averaged dry-bulb temperature (°C) across Kauai for years 1998–2015 with annual means and interannual statistics of monthly values.

**Month**	**1998**	**1999**	**2000**	**2001**	**2002**	**2003**	**2004**	**2005**	**2006**	**2007**	**2008**	**2009**	**2010**	**2011**	**2012**	**2013**	**2014**	**2015**	**Q1**	**Mean**	**Q3**	**Sdev**	**COV (%)**	**Min**	**Max**	**Range**
**January**	23.5	23.5	22.8	24.7	23.6	23.2	24.0	23.8	24.1	24.3	23.3	23.5	23.6	23.9	24.1	23.2	23.6	23.5	23.5	23.7	24.0	0.5	1.90	22.8	24.7	1.9
**February**	23.1	23.2	23.8	23.7	22.6	23.1	24.5	23.3	22.9	23.3	24.0	22.6	22.9	24.1	23.5	22.9	23.6	23.8	23.0	23.4	23.7	0.5	2.28	22.6	24.5	1.9
**March**	24.3	23.3	23.9	23.9	23.0	24.6	23.2	23.1	23.8	24.3	24.7	22.6	23.1	24.0	23.0	22.8	23.4	22.9	23.0	23.6	24.0	0.6	2.74	22.6	24.7	2.1
**April**	23.3	24.0	23.1	24.0	24.9	24.7	24.2	24.4	23.6	24.4	24.5	23.0	23.5	24.9	24.0	24.4	24.0	24.9	23.7	24.1	24.5	0.6	2.49	23.0	24.9	1.9
**May**	23.6	25.3	25.2	25.3	25.7	25.8	25.5	26.0	24.4	25.8	25.8	25.4	25.4	25.1	24.7	25.4	25.8	24.9	25.1	25.3	25.8	0.6	2.34	23.6	26.0	2.4
**June**	24.9	25.4	26.1	25.6	26.6	26.9	26.3	26.5	26.3	26.4	26.2	26.6	26.0	25.4	25.3	26.0	26.3	26.4	25.7	26.1	26.4	0.5	2.04	24.9	26.9	1.9
**July**	25.6	25.7	26.2	26.7	26.5	26.9	27.4	26.6	26.5	26.7	26.8	26.7	26.3	26.0	25.6	26.7	27.1	27.7	26.2	26.5	26.8	0.6	2.20	25.6	27.7	2.2
**August**	26.4	26.3	26.6	26.8	26.9	27.2	27.8	27.3	27.0	27.1	26.9	26.9	26.5	26.2	26.1	27.1	27.5	28.1	26.5	26.9	27.1	0.5	2.03	26.1	28.1	2.0
**September**	26.3	26.2	26.6	26.9	27.2	27.5	27.7	27.2	27.1	26.9	27.0	27.0	26.5	26.4	26.1	27.3	28.2	28.0	26.6	27.0	27.3	0.6	2.23	26.1	28.2	2.1
**October**	25.9	25.7	26.2	26.1	26.6	27.3	27.0	26.3	26.8	26.3	26.4	27.1	26.5	26.1	26.4	26.8	27.6	26.6	26.2	26.5	26.8	0.5	1.86	25.7	27.6	1.9
**November**	25.1	24.7	25.1	25.4	25.5	25.8	26.0	25.6	26.2	25.6	25.5	25.4	25.5	24.8	25.3	25.5	25.7	26.3	25.3	25.5	25.7	0.4	1.66	24.7	26.3	1.6
**December**	23.9	23.9	24.9	24.6	24.6	24.4	24.6	24.8	24.7	24.3	24.6	24.3	24.6	23.9	24.5	24.9	24.0	25.1	24.3	24.5	24.7	0.4	1.52	23.9	25.1	1.3
**Annual Mean**	**24.7**	**24.8**	**25.0**	**25.3**	**25.3**	**25.6**	**25.7**	**25.4**	**25.3**	**25.5**	**25.5**	**25.1**	**25.0**	**25.1**	**24.9**	**25.3**	**25.6**	**25.7**	**25.1**	**25.3**	**25.5**	**0.3**	**1.22**	**24.7**	**25.7**	**1.0**

**Table 16:** Monthly averaged dry-bulb temperature (°C) across Lanai for years 1998–2015 with annual means and interannual statistics of monthly values.

**Month**	**1998**	**1999**	**2000**	**2001**	**2002**	**2003**	**2004**	**2005**	**2006**	**2007**	**2008**	**2009**	**2010**	**2011**	**2012**	**2013**	**2014**	**2015**	**Q1**	**Mean**	**Q3**	**Sdev**	**COV (%)**	**Min**	**Max**	**Range**
**January**	24.4	23.7	23.0	24.6	24.0	24.2	24.6	24.7	24.4	24.6	23.6	23.8	24.7	24.4	24.4	23.7	24.2	24.2	23.8	24.2	24.5	0.5	1.91	23.0	24.7	1.7
**February**	23.9	23.6	24.1	24.0	23.2	23.7	25.1	24.1	23.4	23.7	24.1	22.9	24.0	25.0	24.0	23.1	24.8	24.8	23.6	24.0	24.1	0.6	2.66	22.9	25.1	2.2
**March**	24.8	23.6	24.1	24.1	24.0	25.1	24.3	24.1	24.1	24.7	24.7	22.9	23.8	24.6	23.5	23.7	24.3	24.1	23.9	24.1	24.5	0.5	2.22	22.9	25.1	2.2
**April**	23.7	24.1	23.5	24.2	25.5	25.0	25.0	24.9	23.9	24.9	24.8	23.6	24.3	25.7	24.3	25.3	24.6	25.4	24.1	24.6	25.0	0.7	2.79	23.5	25.7	2.2
**May**	24.0	25.2	25.1	25.2	26.1	25.9	26.1	26.4	24.6	25.8	25.6	26.3	25.6	25.5	25.0	26.1	26.4	25.3	25.2	25.6	26.1	0.7	2.56	24.0	26.4	2.4
**June**	25.2	25.4	26.2	25.5	26.5	26.8	26.7	26.5	26.4	26.7	26.3	26.7	26.0	25.5	25.3	26.1	26.5	26.8	25.7	26.2	26.6	0.5	2.08	25.2	26.8	1.6
**July**	25.9	25.7	26.3	26.6	26.6	26.9	27.6	26.9	26.7	26.8	27.1	26.9	26.3	26.1	25.9	26.9	27.2	28.2	26.3	26.7	26.9	0.6	2.32	25.7	28.2	2.4
**August**	26.6	26.3	26.8	27.0	27.1	27.3	28.0	27.5	27.3	27.2	26.8	27.2	26.5	26.4	26.4	27.2	27.6	28.6	26.7	27.1	27.3	0.6	2.16	26.3	28.6	2.2
**September**	26.5	26.2	26.9	27.0	27.2	27.8	28.0	27.4	27.1	27.1	26.9	27.2	26.6	26.7	26.3	27.4	28.3	28.5	26.7	27.2	27.4	0.7	2.41	26.2	28.5	2.4
**October**	26.1	26.0	26.2	26.4	26.6	27.7	27.1	26.5	27.0	26.5	26.5	27.6	26.5	26.4	26.6	27.0	27.6	27.7	26.4	26.8	27.1	0.6	2.08	26.0	27.7	1.7
**November**	25.1	24.7	25.1	25.8	26.0	25.8	26.5	25.9	26.4	25.8	25.5	25.7	25.6	24.9	25.2	26.0	26.1	26.7	25.3	25.7	26.0	0.5	2.12	24.7	26.7	2.0
**December**	24.1	24.0	25.0	24.6	25.0	24.8	25.1	25.2	24.9	24.6	24.6	25.1	25.1	24.0	24.6	25.5	24.5	25.5	24.6	24.8	25.1	0.5	1.82	24.0	25.5	1.5
**Annual Mean**	**25.0**	**24.9**	**25.2**	**25.4**	**25.7**	**26.0**	**26.2**	**25.9**	**25.5**	**25.7**	**25.6**	**25.5**	**25.4**	**25.4**	**25.1**	**25.7**	**26.0**	**26.3**	**25.4**	**25.6**	**25.8**	**0.4**	**1.51**	**24.9**	**26.3**	**1.4**

**Table 17:** Monthly averaged dry-bulb temperature (°C) across Maui for years 1998–2015 with annual means and interannual statistics of monthly values.

**Month**	**1998**	**1999**	**2000**	**2001**	**2002**	**2003**	**2004**	**2005**	**2006**	**2007**	**2008**	**2009**	**2010**	**2011**	**2012**	**2013**	**2014**	**2015**	**Q1**	**Mean**	**Q3**	**Sdev**	**COV (%)**	**Min**	**Max**	**Range**
**January**	24.5	23.5	22.8	24.4	23.8	24.3	24.6	24.8	24.2	24.4	23.4	23.7	24.7	24.3	24.3	23.6	24.1	24.3	23.7	24.1	24.4	0.5	2.19	22.8	24.8	2.0
**February**	23.8	23.3	23.9	23.8	23.0	23.5	25.0	23.9	23.1	23.6	23.9	22.6	23.9	24.9	23.8	22.8	24.8	24.8	23.4	23.8	23.9	0.7	2.99	22.6	25.0	2.4
**March**	24.6	23.3	23.9	23.8	23.7	25.0	24.1	24.0	24.0	24.7	24.3	22.7	23.6	24.4	23.3	23.6	24.1	23.9	23.6	23.9	24.3	0.6	2.32	22.7	25.0	2.3
**April**	23.5	23.7	23.2	23.8	25.2	24.6	24.7	24.6	23.6	24.5	24.5	23.4	24.0	25.3	23.9	25.2	24.3	25.1	23.8	24.3	24.7	0.7	2.81	23.2	25.3	2.1
**May**	23.7	24.9	24.8	24.8	25.7	25.5	25.9	26.0	24.3	25.4	25.3	26.1	25.3	25.2	24.6	25.8	26.2	25.0	24.8	25.2	25.8	0.7	2.63	23.7	26.2	2.5
**June**	25.0	25.0	25.8	25.1	26.1	26.3	26.3	26.1	25.9	26.2	25.8	26.3	25.6	25.2	25.0	25.8	26.1	26.4	25.3	25.8	26.2	0.5	1.97	25.0	26.4	1.4
**July**	25.6	25.4	26.0	26.2	26.2	26.5	27.2	26.5	26.3	26.4	26.6	26.5	25.9	25.7	25.5	26.5	26.8	27.8	26.0	26.3	26.5	0.6	2.29	25.4	27.8	2.4
**August**	26.4	26.0	26.5	26.6	26.7	27.0	27.7	27.1	26.9	26.8	26.4	26.9	26.1	26.1	26.0	26.8	27.2	28.3	26.4	26.8	27.0	0.6	2.25	26.0	28.3	2.4
**September**	26.2	25.8	26.6	26.6	26.8	27.5	27.6	27.1	26.7	26.7	26.5	26.8	26.2	26.2	25.9	27.0	28.0	28.3	26.3	26.8	27.1	0.7	2.55	25.8	28.3	2.5
**October**	25.8	25.8	25.9	26.1	26.3	27.5	26.9	26.1	26.8	26.2	26.2	27.2	26.2	26.0	26.3	26.8	27.3	27.5	26.1	26.5	26.9	0.6	2.21	25.8	27.5	1.8
**November**	24.9	24.4	24.9	25.6	25.8	25.6	26.3	25.8	26.2	25.6	25.3	25.4	25.4	24.7	25.0	25.8	25.9	26.5	25.1	25.5	25.8	0.6	2.25	24.4	26.5	2.1
**December**	23.9	24.0	24.8	24.5	24.8	24.6	24.9	25.1	24.7	24.4	24.4	25.1	25.0	23.8	24.4	25.2	24.4	25.3	24.4	24.6	25.0	0.5	1.83	23.8	25.3	1.5
**Annual Mean**	**24.8**	**24.6**	**24.9**	**25.1**	**25.4**	**25.7**	**25.9**	**25.6**	**25.2**	**25.4**	**25.2**	**25.2**	**25.2**	**25.1**	**24.9**	**25.4**	**25.8**	**26.1**	**25.1**	**25.3**	**25.6**	**0.4**	**1.58**	**24.6**	**26.1**	**1.5**

**Table 18:** Monthly averaged dry-bulb temperature (°C) across Moloka׳i for years 1998–2015 with annual means and interannual statistics of monthly values.

**Month**	**1998**	**1999**	**2000**	**2001**	**2002**	**2003**	**2004**	**2005**	**2006**	**2007**	**2008**	**2009**	**2010**	**2011**	**2012**	**2013**	**2014**	**2015**	**Q1**	**Mean**	**Q3**	**Sdev**	**COV (%)**	**Min**	**Max**	**Range**
**January**	24.2	23.5	22.9	24.5	23.8	24.0	24.4	24.6	24.3	24.5	23.4	23.7	24.5	24.2	24.3	23.6	24.0	24.0	23.7	24.0	24.4	0.5	1.92	22.9	24.6	1.7
**February**	23.7	23.4	23.9	23.8	23.0	23.5	25.0	23.9	23.2	23.6	24.0	22.7	23.8	24.8	23.9	22.9	24.6	24.5	23.4	23.8	24.0	0.6	2.67	22.7	25.0	2.3
**March**	24.6	23.4	23.9	23.9	23.7	24.9	24.0	23.9	24.0	24.5	24.6	22.8	23.6	24.5	23.4	23.4	24.1	23.8	23.7	23.9	24.4	0.5	2.24	22.8	24.9	2.2
**April**	23.5	23.9	23.3	24.0	25.3	24.9	24.8	24.7	23.7	24.6	24.6	23.4	24.0	25.5	24.2	25.2	24.4	25.2	23.9	24.4	24.8	0.7	2.82	23.3	25.5	2.3
**May**	23.8	25.0	25.0	25.0	25.9	25.7	25.9	26.2	24.4	25.6	25.5	26.0	25.5	25.4	24.8	25.9	26.3	25.0	25.0	25.4	25.9	0.6	2.55	23.8	26.3	2.5
**June**	25.0	25.3	26.0	25.4	26.4	26.6	26.5	26.3	26.2	26.5	26.1	26.5	25.9	25.4	25.2	26.0	26.3	26.6	25.5	26.0	26.5	0.5	2.03	25.0	26.6	1.6
**July**	25.7	25.6	26.2	26.5	26.4	26.7	27.5	26.7	26.4	26.6	26.8	26.7	26.2	25.9	25.7	26.7	27.0	28.0	26.2	26.5	26.7	0.6	2.31	25.6	28.0	2.4
**August**	26.4	26.2	26.6	26.8	27.0	27.1	27.9	27.3	27.1	27.0	26.7	27.0	26.3	26.3	26.2	27.1	27.5	28.4	26.5	26.9	27.1	0.6	2.14	26.2	28.4	2.2
**September**	26.3	26.0	26.7	26.8	27.0	27.6	27.8	27.2	26.9	26.9	26.7	27.0	26.5	26.5	26.1	27.3	28.2	28.3	26.6	27.0	27.3	0.7	2.43	26.0	28.3	2.3
**October**	25.9	25.9	26.1	26.2	26.5	27.6	27.0	26.3	26.9	26.4	26.4	27.4	26.4	26.2	26.5	26.9	27.5	27.4	26.3	26.6	27.0	0.5	2.04	25.9	27.6	1.7
**November**	25.0	24.6	25.0	25.6	25.8	25.7	26.3	25.7	26.2	25.7	25.4	25.6	25.5	24.8	25.1	25.9	25.9	26.6	25.2	25.6	25.8	0.5	2.08	24.6	26.6	2.0
**December**	24.0	23.9	24.9	24.6	24.9	24.7	25.0	25.0	24.8	24.5	24.5	24.9	24.9	23.9	24.5	25.3	24.4	25.4	24.5	24.7	24.9	0.4	1.76	23.9	25.4	1.5
**Annual Mean**	**24.9**	**24.7**	**25.0**	**25.3**	**25.5**	**25.8**	**26.0**	**25.7**	**25.4**	**25.5**	**25.4**	**25.3**	**25.3**	**25.3**	**25.0**	**25.5**	**25.8**	**26.1**	**25.3**	**25.4**	**25.6**	**0.4**	**1.49**	**24.7**	**26.1**	**1.4**

**Table 19:** Monthly averaged dry-bulb temperature (°C) across Ni׳ihau for years 1998–2015 with annual means and interannual statistics of monthly values.

**Month**	**1998**	**1999**	**2000**	**2001**	**2002**	**2003**	**2004**	**2005**	**2006**	**2007**	**2008**	**2009**	**2010**	**2011**	**2012**	**2013**	**2014**	**2015**	**Q1**	**Mean**	**Q3**	**Sdev**	**COV (%)**	**Min**	**Max**	**Range**
**January**	23.3	23.5	22.9	24.6	23.7	23.0	24.0	23.8	24.2	24.3	23.4	23.5	23.4	24.0	24.0	23.3	23.5	23.3	23.3	23.7	24.0	0.5	1.97	22.9	24.6	1.7
**February**	22.9	23.2	23.7	23.6	22.7	23.1	24.5	23.4	23.0	23.3	23.8	22.7	22.6	24.0	23.4	22.9	23.4	23.5	22.9	23.3	23.6	0.5	2.10	22.6	24.5	1.8
**March**	24.0	23.3	23.8	23.8	22.9	24.2	23.3	23.0	23.9	24.0	24.6	22.6	22.9	24.1	23.0	22.6	23.2	22.8	22.9	23.4	23.9	0.6	2.60	22.6	24.6	2.0
**April**	23.3	23.9	23.2	24.1	24.5	24.7	24.1	24.3	23.7	24.2	24.3	22.8	23.4	24.7	23.9	24.1	23.9	24.7	23.7	24.0	24.3	0.5	2.24	22.8	24.7	1.9
**May**	23.6	25.1	25.0	25.1	25.6	25.6	25.3	25.8	24.3	25.5	25.5	24.7	25.1	25.0	24.5	25.0	25.3	24.8	24.8	25.1	25.4	0.5	2.16	23.6	25.8	2.2
**June**	24.9	25.3	25.9	25.6	26.5	26.6	26.3	26.4	26.1	26.3	26.1	26.4	25.8	25.4	25.1	25.8	26.1	26.2	25.7	25.9	26.3	0.5	1.92	24.9	26.6	1.7
**July**	25.4	25.6	26.1	26.6	26.4	26.7	27.3	26.5	26.3	26.6	26.7	26.6	26.1	25.9	25.4	26.5	26.9	27.5	26.1	26.4	26.7	0.6	2.15	25.4	27.5	2.1
**August**	26.2	26.2	26.5	26.7	26.8	27.0	27.7	27.0	26.8	27.0	26.8	26.8	26.3	26.1	25.9	26.9	27.3	27.9	26.4	26.8	27.0	0.5	1.97	25.9	27.9	2.0
**September**	26.2	26.1	26.6	26.8	27.0	27.3	27.7	27.2	27.1	26.9	26.8	26.9	26.3	26.2	25.9	27.1	27.9	27.9	26.4	26.9	27.2	0.6	2.19	25.9	27.9	2.0
**October**	25.8	25.7	26.2	26.1	26.5	27.1	26.9	26.4	26.6	26.3	26.3	26.8	26.4	26.0	26.1	26.5	27.6	26.4	26.1	26.4	26.6	0.5	1.73	25.7	27.6	1.8
**November**	25.1	24.8	25.2	25.2	25.5	25.8	26.0	25.6	26.1	25.5	25.4	25.5	25.5	24.9	25.2	25.4	25.7	26.2	25.2	25.5	25.7	0.4	1.51	24.8	26.2	1.4
**December**	24.0	24.0	24.8	24.6	24.5	24.5	24.7	24.6	24.7	24.4	24.7	24.1	24.6	24.0	24.5	24.8	24.0	25.1	24.2	24.5	24.7	0.3	1.35	24.0	25.1	1.1
**Annual Mean**	**24.6**	**24.7**	**25.0**	**25.2**	**25.2**	**25.5**	**25.7**	**25.3**	**25.2**	**25.4**	**25.4**	**25.0**	**24.9**	**25.0**	**24.8**	**25.1**	**25.4**	**25.5**	**25.0**	**25.2**	**25.4**	**0.3**		**24.6**	**25.7**	**1.1**

**Table 20:** Monthly averaged dry-bulb temperature (°C) across O׳ahu for years 1998–2015 with annual means and interannual statistics of monthly values.

**Month**	**1998**	**1999**	**2000**	**2001**	**2002**	**2003**	**2004**	**2005**	**2006**	**2007**	**2008**	**2009**	**2010**	**2011**	**2012**	**2013**	**2014**	**2015**	**Q1**	**Mean**	**Q3**	**Sdev**	**COV (%)**	**Min**	**Max**	**Range**
**January**	24.1	23.5	22.8	24.9	23.9	23.9	24.3	24.4	24.3	24.5	23.5	23.7	24.4	24.2	24.6	23.6	24.1	24.1	23.8	24.0	24.4	0.5	2.03	22.8	24.9	2.1
**February**	23.7	23.5	24.2	24.0	23.0	23.6	25.0	23.8	23.2	23.6	24.3	22.9	23.7	24.8	24.0	23.1	24.5	24.6	23.5	23.9	24.2	0.6	2.62	22.9	25.0	2.2
**March**	24.9	23.6	24.1	24.2	23.7	25.2	23.9	23.8	24.0	24.8	25.1	22.9	23.7	24.5	23.5	23.5	24.2	23.8	23.7	24.1	24.4	0.6	2.55	22.9	25.2	2.3
**April**	23.6	24.2	23.4	24.3	25.7	25.1	24.8	24.9	23.8	24.9	24.8	23.6	24.1	25.7	24.5	25.3	24.6	25.4	24.1	24.6	25.0	0.7	2.90	23.4	25.7	2.3
**May**	23.9	25.4	25.3	25.5	26.0	26.2	26.1	26.4	24.7	26.2	26.0	26.4	25.8	25.5	25.2	26.2	26.6	25.4	25.4	25.7	26.2	0.7	2.62	23.9	26.6	2.7
**June**	25.2	25.6	26.3	25.6	26.8	27.2	26.6	26.6	26.7	26.8	26.5	26.9	26.3	25.6	25.6	26.3	26.6	27.0	25.8	26.4	26.8	0.6	2.21	25.2	27.2	2.0
**July**	25.9	25.8	26.4	26.9	26.7	27.1	27.8	27.0	26.9	26.9	27.1	27.0	26.6	26.3	26.0	27.0	27.3	28.2	26.4	26.8	27.1	0.6	2.30	25.8	28.2	2.4
**August**	26.7	26.5	26.8	27.1	27.2	27.4	28.1	27.7	27.3	27.4	27.0	27.2	26.7	26.7	26.5	27.4	27.8	28.6	26.7	27.2	27.4	0.6	2.08	26.5	28.6	2.0
**September**	26.5	26.3	26.9	27.1	27.5	28.0	28.0	27.4	27.3	27.1	27.2	27.4	26.9	27.0	26.4	27.5	28.5	28.4	26.9	27.3	27.5	0.6	2.30	26.3	28.5	2.2
**October**	26.1	26.0	26.3	26.4	26.7	27.8	27.2	26.5	27.1	26.6	26.7	27.7	26.8	26.5	26.8	27.1	27.7	27.2	26.5	26.8	27.2	0.5	1.99	26.0	27.8	1.8
**November**	25.2	24.6	25.2	25.7	25.9	25.9	26.3	25.9	26.4	25.7	25.7	25.6	25.7	24.9	25.5	25.9	26.0	26.6	25.5	25.7	25.9	0.5	1.97	24.6	26.6	2.0
**December**	24.0	23.9	25.2	24.7	25.0	24.7	24.9	25.2	24.9	24.5	24.6	24.8	24.9	24.0	24.7	25.3	24.4	25.6	24.5	24.7	25.0	0.5	1.86	23.9	25.6	1.7
**Annual Mean**	**25.0**	**24.9**	**25.2**	**25.5**	**25.7**	**26.0**	**26.1**	**25.8**	**25.6**	**25.8**	**25.7**	**25.5**	**25.5**	**25.5**	**25.3**	**25.7**	**26.0**	**26.2**	**25.5**	**25.6**	**25.8**	**0.4**	**1.42**	**24.9**	**26.2**	**1.3**

## References

[1] State of Hawaii, Hawaii Clean Energy Initiative, 2018. 〈http://www.hawaiicleanenergyinitiative.org/〉, (Accessed 03 January 2018).

[2] A. Habte, M. Sengupta, A. Lopez, Evaluation of the National Solar Radiation Database (NSRDB): 1998–2015, Golden, CO, 2017. 〈http://www.nrel.gov/docs/fy17osti/67722.pdf〉.

[3] Rienecker M.M., Suarez M.J., Gelaro R., Todling R., Bacmeister J., Liu E., Bosilovich M.G., Schubert S.D., Takacs L., Kim G.K., Bloom S., Chen J., Collins D., Conaty A., Da Silva A., Gu W., Joiner J., Koster R.D., Lucchesi R., Molod A., Owens T., Pawson S., Pegion P., Redder C.R., Reichle R., Robertson F.R., Ruddick A.G., Sienkiewicz M., Woollen J. (2011). MERRA: NASA׳s modern-era retrospective analysis for research and applications. J. Clim..

[4] Pfeifroth U., Mueller R. (2012). Evaluation of satellite-based and reanalysis precipitation data in the tropical Pacific. J. Appl. Meteorol. Climatol..

[5] Yi Y., Kimball J.S., Jones L.A., Reichle R.H., Mcdonald K.C. (2011). Evaluation of MERRA land surface estimates in preparation for the soil moisture active passive mission. J. Clim..

[6] Jones R.W., Renfrew I.A., Orr A., Webber B.G.M., Holland D.M., Lazzara M.A. (2016). Evaluation of four global reanalysis products using in situ observations in the Amundsen Sea Embayment, Antarctica. J. Geophys. Res. Atmos..

[7] R. Bryce, I. Losada Carreño, A.Kumler, B.-M. Hodge, B. Roberts and C. Brancucci Martinez-Anido, Consequences of neglecting the interannual variability of the solar resource: A case study of photovoltaic power among the Hawaiian Islands. Solar Energy, 167 (2018) 61-75. 10.1016/j.solener.2018.03.085.

[8] Lave M., Kleissl J. (2010). Solar variability of four sites across the state of Colorado. Renew. Energy.

