# Supplementary material for: Annually and monthly resolved solar irradiance and atmospheric temperature data across the Hawaiian archipelago from 1998 to 2015 with interannual summary statistics
Source: Data Brief. 2018 May 23;19:896–920. doi: 10.1016/j.dib.2018.05.099 (PMC5997942; doi:10.1016/j.dib.2018.05.099)
Supplement: Supplementary file 1 — Supplementary material [file mmc1.docx]

**Data Article**

**Title**: Annually and monthly resolved solar irradiance and atmospheric temperature data across the Hawaiian archipelago from 1998-2015 with interannual summary statistics

**Authors**: Richard Bryce^a,b^, Ignacio Losada Carreño^a,c^, Andrew Kumler^a^, Bri-Mathias Hodge^a^, Billy Roberts^a^, Carlo Brancucci Martinez-Anido^a^

**Affiliations**:

*^a^ National Renewable Energy Laboratory, Golden Colorado*

*^b^ Department of Mechanical and Industrial Engineering, University of Massachusetts, Amherst Massachusetts*

*^c^ Department of Mechanical Engineering, Northern Arizona University, Flagstaff Arizona*

**Contact email**: Carlo.Brancucci@nrel.gov

**Declaration of Conflicts of Interest:** There are no potential conflicts of interest.
